# CRISPR–Cas technologies in neurodegenerative disorders: mechanistic insights, therapeutic potential, and translational challenges

**DOI:** 10.3389/fneur.2025.1737468

**Published:** 2026-01-27

**Authors:** Raya Kh. Yashooa, Ari Q. Nabi, Shukur Wasman Smail, Sarkar Sardar Azeez, Wissam Albeer Nooh, Suhad A. Mustafa, Abd Al-Bar Al-Farha, Nazzareno Capitanio, Mudhir Sabir Shekha

**Affiliations:** 1 Department of Biology, College of Education for Pure Sciences, University of Al-Hamdaniya, Mosul, Iraq; 2 Department of Biology, College of Science, Salahaddin University-Erbil, Erbil, Iraq; 3 College of Pharmacy, Cihan University-Erbil, Erbil, Iraq; 4 Department of Medical Laboratory Technology, Soran Technical College, Erbil Polytechnic University, Erbil, Iraq; 5 General Directorate of Scientific Research Center, Salahaddin University-Erbil, Erbil, Iraq; 6 Department of Biotechnology and Food Sciences, Technical Agricultural College-Mosul, Northern Technical University, Mosul, Iraq; 7 Department of Clinical and Experimental Medicine, University of Foggia, Foggia, Italy; 8 Department of Medical Cell Biology, Uppsala University, Uppsala, Sweden

**Keywords:** applications, challenges, CRISPR–Cas, neurodegenerative disorders, therapeutic strategies

## Abstract

CRISPR–Cas genome-editing technologies have emerged as powerful tools for precise DNA and RNA modulation, offering promising therapeutic strategies for neurodegenerative disorders such as Alzheimer's disease (AD), Parkinson's disease (PD), Huntington's disease (HD), and amyotrophic lateral sclerosis (ALS). This review critically evaluates current CRISPR/Cas applications in neurodegeneration, with emphasis on mechanistic insights, therapeutic outcomes, and translational feasibility. Preclinical and early translational studies demonstrate that CRISPR–Cas platforms can correct pathogenic mutations, suppress toxic gene expression, and restore neuronal function. Advanced modalities, including base and prime editing, CRISPRi/a, and RNA-targeting Cas systems, improve precision and reduce genomic damage, which is particularly advantageous in post-mitotic neurons. Emerging CRISPR-based diagnostics (e.g., SHERLOCK and DETECTR), AI-assisted sgRNA design, and machine-learning approaches for predicting off-target effects further enhance the safety, stratification, and monitoring of CRISPR therapeutics. In parallel, patient-derived brain organoids and assembloids provide scalable human-relevant platforms for mechanistic studies and preclinical validation. Despite this progress, major challenges remain, including efficient delivery across the blood–brain barrier, immune responses, long-term safety, and ethical and regulatory considerations. Overall, CRISPR–Cas technologies hold strong potential as disease-modifying interventions for neurodegenerative disorders, provided that advances in delivery systems, artificial intelligence integration, and regulatory oversight continue to evolve toward clinical translation.

## Graphical Abstract

**Figure:**
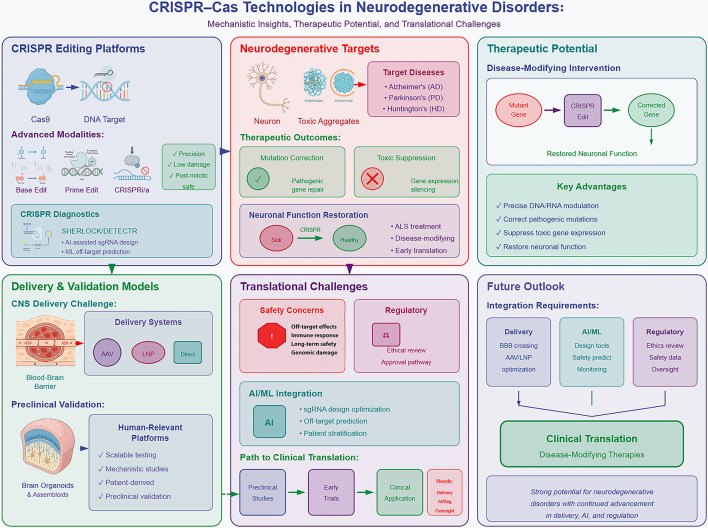

## 1 Introduction

Neurodegenerative disorders (NDs) are a subset of neurological disorders characterized by progressive and irreversible neuronal loss, leading to cognitive, motor, or functional impairments (1). According to the Global Burden of Disease Study (GBD), NDs are the leading cause of disability-adjusted life years (DALYs) and the second leading cause of death worldwide. In 2016, over 276 million DALYs were attributed to NDs, with a significant proportion due to neurodegenerative diseases. These conditions not only reduce life expectancy but also impose an immense strain on healthcare systems, families, and caregivers (2).

Some of the most prevalent NDs include Alzheimer's disease (AD), Parkinson's disease (PD), Huntington's disease (HD), and amyotrophic lateral sclerosis (ALS) (3, 4). The pathophysiology of these disorders is complex, often involving multifactorial genetic and environmental interactions that contribute to neuroinflammation, protein misfolding, mitochondrial dysfunction, and synaptic dysregulation (5).

Despite extensive research, developing disease-modifying treatments for major NDs remains a formidable challenge. For AD, approved therapies (acetylcholinesterase inhibitors, memantine, and recent anti-amyloid antibodies) offer modest symptomatic benefit or, at best, a modest slowing of clinical decline, without halting the underlying pathological cascade (6, 7). PD management relies primarily on dopamine replacement (e.g., L-DOPA) and deep brain stimulation, which effectively manage motor symptoms but do not address the progressive neurodegeneration (8, 9). HD, a monogenic disorder, has no disease-modifying treatment; current care is purely supportive (10, 11).

This critical therapeutic gap stems from several intrinsic obstacles. First, the complex, multifactorial pathogenesis of NDs involves diverse genetic susceptibilities, proteinopathies, and dysfunctional cellular pathways, making single-target pharmacological interventions often insufficient (12). Second, therapeutic delivery to the central nervous system (CNS) is profoundly constrained by the highly selective blood–brain barrier (BBB). Rather than absolute exclusion, the BBB employs tight junctions, efflux transporters (e.g., P-glycoprotein), metabolic enzymes, and receptor-mediated trafficking to regulate xenobiotic entry based on molecular size, charge, lipophilicity, and carrier availability. Consequently, only ~2%−5% of small molecules and virtually no unmodified biologics reach therapeutically relevant brain concentrations without specialized delivery strategies (13, 14). Third, the limited intrinsic regenerative capacity of central neurons, coupled with late clinical detection and heterogeneous progression rates, significantly narrows the therapeutic window for disease-modifying interventions (15).

Given this therapeutic stagnation, genome-editing technologies offer the unprecedented ability to intervene at the molecular origins of neurodegeneration (16). Many NDs possess well-validated genetic drivers that represent rational clustered regularly interspaced short palindromic repeats (CRISPR) targets, including *huntingtin (HTT)* CAG repeat expansions in *HD, Presenilin-1 (PSEN1)/PSEN2* and *amyloid precursor protein (APP)* mutations in familial AD (fAD), and *alpha-synuclein (SNCA)* and *leucine-rich repeat kinase 2 (LRRK2)* in PD (17–19). Thus, the genetic tractability of major NDs provides a compelling mechanistic rationale for genome editing as a disease-modifying therapeutic strategy.

The discovery of CRISPR-associated protein (Cas) technology has transformed biomedical research, offering precise and programmable tools to modify the genome and transcriptome. Classical Cas9 enables targeted gene knockout, correction, or insertion via guide RNA-directed cleavage (20). The CRISPR toolbox has rapidly expanded to encompass a range of complementary modalities. Catalytically inactive (dead) Cas 9 (dCas9) allows transcriptional repression or activation without altering the DNA sequence (CRISPR interference (CRISPRi/CRISPR activation (CRISPRa), base and prime editors facilitate single-nucleotide resolution corrections, and RNA-targeting Cas13 systems permit transient modulation of disease-associated transcripts (21–24). These diverse strategies are particularly advantageous in post-mitotic neurons, where permanent DNA breaks carry increased risk, and reversible or finely tuned modulation of gene expression or pathogenic transcripts may provide safer, more effective interventions. Neurons have intrinsically limited capacity for homology-directed repair (HDR), meaning that double-stranded breaks (DSBs) introduced by conventional nucleases can result in persistent genomic damage and cytotoxicity (25, 26).

By leveraging these diverse strategies, CRISPR technologies provide the ability not only to correct causative mutations but also to modulate pathogenic pathways, restore cellular homeostasis, and complement conventional symptomatic therapies.

Despite these advances, significant hurdles remain, including efficient delivery across the BBB, off-target effects, immune activation, and ethical considerations. Addressing these barriers is essential to realizing the full therapeutic potential of genome editing in NDs (27).

This review aims to explore the potential applications of CRISPR–Cas technology in the treatment of neurodegenerative disorders, examining its mechanisms and therapeutic possibilities across a range of conditions. Additionally, we will investigate the challenges in applying CRISPR–Cas technology to neurodegenerative disorders, including delivery barriers, off-target effects, immune responses, and ethical concerns, while proposing potential strategies to overcome these obstacles. Finally, we will examine the status of CRISPR-based therapeutics in clinical trials, identify gaps, and propose future directions toward the successful application of CRISPR–Cas in neurodegenerative diseases.

## 2 CRISPR–Cas system

### 2.1 An adaptive immune system of bacteria and archaea

The CRISPR–Cas system serves as an adaptive immune defense mechanism in bacteria and archaea, protecting against invading genetic elements such as bacteriophages and plasmids (28). This system operates through three main stages: adaptation, expression, and interference. During adaptation, foreign DNA fragments, known as protospacers, are incorporated into the CRISPR array as spacers. In the expression stage, the CRISPR array is transcribed into a precursor CRISPR RNA (pre-crRNA), which is then processed into mature crRNAs. Finally, in the interference stage, the mature crRNA directs Cas proteins, which function as endonucleases, to recognize the complementary sequences of the invading genetic element and create DSBs, thereby neutralizing the threat (29, 30).

### 2.2 CRISPR-Cas systems and classes

The CRISPR–Cas system is categorized into two major classes, Class 1 and Class 2, encompassing six types and 33 subtypes. Class 1 is characterized by multiprotein effector complexes and is prevalent in both bacteria and archaea, whereas Class 2, found exclusively in bacteria, relies on a single multifunctional effector protein (31). Each class is further divided into three types: Class 1 includes Types I, III, and IV, whereas Class 2 comprises Types II, V, and VI.

The Class 1 CRISPR–Cas system, particularly Type I, employs multiple Cas proteins that interact with crRNA to form the CRISPR-associated complex for antiviral defense (Cascade). Cascade binds to foreign DNA, allowing crRNA to hybridize with the complementary strand, forming an R-loop, which is then cleaved by Cas3, an enzyme with helicase and nuclease activity (32). The Type I system is the most prevalent among prokaryotes and has further subtypes, such as Type I-A to I-F, each exhibiting distinct Cas protein compositions and mechanisms of interference (33). Similarly, Type III systems, which use Cas10 as the signature protein, exhibit RNA and DNA cleavage capabilities, often functioning through a co-transcriptional targeting mechanism that recognizes RNA and degrades DNA in a coordinated manner (34). Type IV systems, though less characterized, are generally found in plasmids rather than chromosomal CRISPR loci and exhibit a minimal set of Cas proteins, including Cas12a [formerly CRISPR-associated protein 1 (Csf1)]. Their exact function remains under investigation, though they appear to contribute to horizontal gene transfer regulation (35).

In contrast, Class 2 CRISPR–Cas systems rely on single-effector proteins, such as Cas9 in Type II, Cas12 in Type V, and Cas13 in Type VI (36). Cas9, the most well-studied and widely utilized in genome editing, is guided by a single-guide RNA (sgRNA) that directs site-specific DNA cleavage at the protospacer (37). Type II CRISPR systems require a trans-activating CRISPR RNA (tracrRNA) to assist in crRNA maturation and target cleavage (38). Type V systems, which include Cas12 proteins, differ from Cas9 by generating staggered double-strand DNA breaks rather than blunt-ended cuts. Upon specific target DNA recognition, Cas12 undergoes conformational activation that triggers collateral, nonspecific degradation of surrounding single-stranded DNA (ssDNA) molecules. This property forms the mechanistic basis of CRISPR-based diagnostic platforms, where collateral ssDNA cleavage amplifies signal following accurate target detection (39). Type VI systems, featuring Cas13 effectors, uniquely target RNA instead of DNA. After binding to a complementary RNA substrate, Cas13 becomes catalytically activated and induces indiscriminate collateral cleavage of nearby RNA molecules, enabling programmable transcriptome editing as well as highly sensitive RNA diagnostics (40). The diversity of CRISPR–Cas systems highlights their evolutionary innovation and functional specialization in prokaryotic adaptive immunity.

Despite their prevalence in nature and mechanistic diversity, Class 1 CRISPR–Cas systems remain significantly underrepresented in genome-editing applications. Their effector function relies on large, multi-subunit complexes, which introduces major challenges for clinical translation, particularly regarding delivery into mammalian cells, vector packaging constraints, and protein complex assembly fidelity in heterologous systems (32, 41). Additionally, incomplete characterization of many Class 1 subtypes limits precise manipulation and safety assessments, further constraining their therapeutic development compared with Class 2 systems, where single-effector nucleases such as Cas9 and Cas12 are inherently more compatible with current delivery modalities and regulatory expectations (42).

### 2.3 Genome editing tools: CRISPR-Cas9 and beyond

The CRISPR–Cas9 system has revolutionized genome editing due to its high precision and programmability. The breakthrough realization that CRISPR could be repurposed as a genome-editing tool came from the work of Doudna and Charpentier, who demonstrated that the Cas9 protein could be programmed with a synthetic sgRNA to target specific DNA sequences. Their findings laid the foundation for programmable genome modifications, allowing precise genetic alterations in diverse organisms (20, 43). This system consists of two primary components: the Cas9 endonuclease and a guide RNA (gRNA) (44). The Cas9 protein, originally derived from *Streptococcus pyogenes* (spCas9), is a multidomain endonuclease responsible for generating targeted DSBs in DNA, earning it the designation of a “genetic scissor.” Cas9 comprises two distinct regions: the recognition lobe (REC) and the nuclease lobe (NUC). The NUC region contains the His-Asn-His (HNH) and RuvC nuclease domains, which are responsible for cleaving the complementary and non-complementary DNA strands, respectively, as well as the protospacer adjacent motif (PAM)-interacting domain, which is essential for target DNA recognition (45).

**Figure 1:**
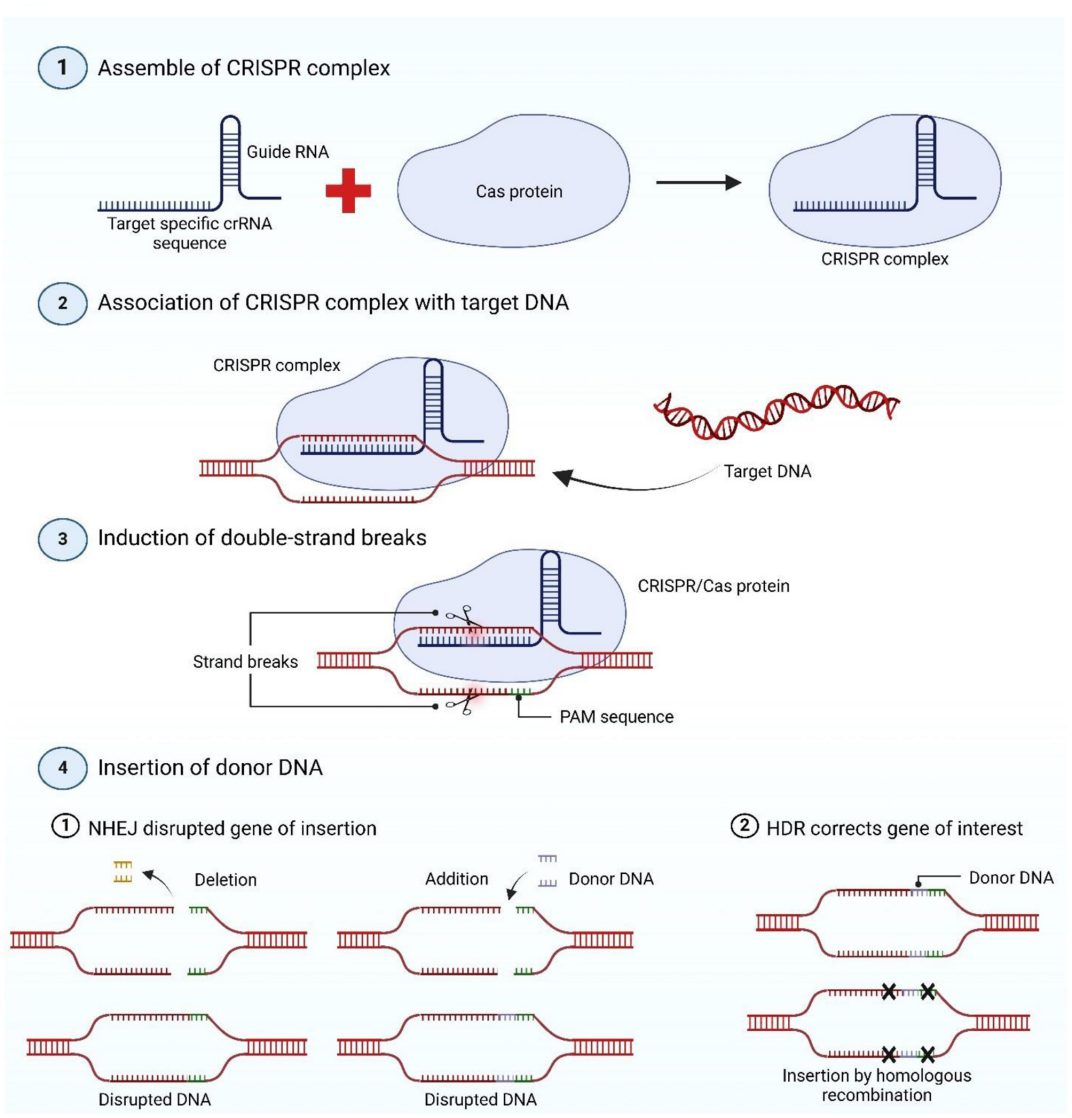
The schematic diagram represents the mechanism of CRISPR–Cas genome editing. An sgRNA is necessary for CRISPR–Cas genome editing because it guides the Cas endonuclease to a particular area of the genomic DNA, causing a double-strand break. Transgenic DNA can be produced by supplying donor DNA in trans, but without donor DNA, the host cell will repair the double-strand break, leading to an insertion or deletion that may disturb a gene's open reading frame. The figure was designed with BioRender (http://Biorender.com/). CRISPR, clustered regularly interspaced short palindromic repeats; Cas, CRISPR-associated protein; crRNA, CRISPR RNA; gRNA, guide RNA; DNA, deoxyribonucleic acid; PAM, protospacer adjacent motif; NHEJ, non-homologous end joining; HDR, homology-directed repair.

The CRISPR–Cas9 genome editing process follows three key steps: target recognition, DNA cleavage, and repair. The gRNA comprises two essential components: a tracrRNA and a crRNA that guides the Cas9 enzyme to a specific target site within the genome (46). Target recognition occurs when the5′ end of the crRNA hybridizes with a complementary sequence in the genome, while the adjacent PAM sequence facilitates Cas9 binding. Upon successful pairing, Cas9 induces a DSB at the target locus, activating the cellular DNA repair machinery (47). Following DSB formation, cells repair the damage through either non-homologous end joining (NHEJ) or HDR pathways. NHEJ is an error-prone mechanism that directly ligates DNA ends, often introducing insertions or deletions (indels) that can disrupt gene function (48). In contrast, HDR enables precise genome modifications by utilizing a homologous DNA template, facilitating accurate base substitutions or insertions (Figure 1) (49).

Substantial technological refinements have enhanced the specificity and versatility of Cas9. High-fidelity variants such as enhanced specificity Cas9 (eSpCas9) and *Streptococcus pyogenes* Cas9–High-Fidelity 1 (SpCas9-HF1) have been engineered to minimize off-target cleavage, improving editing accuracy (50). Cas9 nickases (nCas9), which generate single-strand breaks instead of DSBs, further reduce genomic instability while enabling precise editing (51).

Base editors, such as cytosine base editors (CBEs) and adenine base editors (ABEs), enable targeted single-nucleotide modifications, reducing the risk of undesired genomic alterations (52, 53). Base editing is a kind of genome editing that can change a base pair at the target genomic locus directly and irreversibly without the need for donor templates, DSBs, or HDR procedures 41. Two parts make into DNA base editors: a single-stranded DNA modifying enzyme to target nucleotide alteration and a Cas enzyme for DNA binding. There are now two types of DNA base editors available: CBEs and ABEs. Therefore, four transition mutations (cytosine convent into thymine C → T, T → C, guanine into adenine G → A, and A → G) can be installed using base editing (23, 54). Cytosine is changed into uracil by the first base editor (CBE1) deaminating the exocyclic amine. Uracil's detection by cell replication machinery as a thymine, which resulting transition the C–G to T–A (55). Cas proteins are unrelated to gRNA's activity and rely on it for DNA binding. Therefore, by altering their amino acids or changing their structural makeup, one or both of its domains can be rendered inactive with no loss in their ability to bind. By applying this theory, researchers have created modified Cas endonuclease, resulting enable to disrupt the activation of Ruvc and HNH domains to create as nCas or a dCas. All Cas proteins, including dCas and nCas, are able to merge with various other molecules, such as enzymes, without affecting their ability to bind and cut independently (56).

Prime editing, which employs an engineered reverse transcriptase fused to Cas9 nickase, facilitates the precise insertion or deletion of genetic sequences without requiring donor templates, thereby increasing editing efficiency and accuracy (54). The prime editing method was developed by Anzalone in 2019, which decreases the number of inadvertent errors (54, 57). Researchers demonstrated that prime editing effectively installs all 12 potential base-to-base transformations without requiring donor templates of DNA or inducing DSBs in the target sequence, because this method doesn't depend on DSB 46. It consists of a prime editing guide RNA (pegRNA) and Cas9 (H840A) nickase; the pegRNA is longer than gRNA. The engineered reverse transcriptase (RT) results from fusing pegRNA and Cas9 (H840A) nickase. RT serves as the enzyme that uses the pegRNA as a template to synthesize the edited DNA sequence during prime editing. The prime editing has been designed and screened in human cells. There are prime editing systems that process by incorporating Cas9 (H840A) nickase and RT enzyme, and mutations are introduced when transversions occur (58–60). pegRNA includes tracrRNA and spacer, in addition to gRNA, gRNA connected to a specific gene of RNA sequence, which consists of a primer binding site (PBS), the PBS site contains a complementary sequence to the target region of the edited sequence of DNA. Once the target DNA is bound by the CRISPR–Cas9 system, nCas9 cuts the opposing DNA strand to create a single-stranded break (SSB) in the DNA. Using Cas9 fused to RT, the SSB of the DNA sequence links to the PBS site in the sgRNA, acting as a primer to create a new DNA fragment. The targeted DNA will subsequently be replaced by this freshly created DNA (56).

RNA-targeting CRISPR systems (Cas13 and newer RNA effectors) allow for programmable changes to transcripts without changing DNA permanently. This is useful for reversible changes, antiviral strategies, and diagnostics. However, RNA editors have their own problems, such as collateral cleavage in some cases, activity that depends on the sequence, and the need for repeated dosing for therapeutic effects. Newer engineered variants, on the other hand, show better specificity and less collateral activity (61). Table 1 shows some strengths and challenges of contemporary CRISPR editing modalities.

**Table 1:** The strengths and challenges of contemporary CRISPR editing modalities.

**Platform**	**Strengths**	**Key limitations/challenges**	**Typical use-cases**
Cas9 nuclease	High editing efficiency for knockouts; well-established	DSBs → indels, large deletions/translocations; off-targets; PAM constraints; delivery & immunogenicity	Gene knockout, HDR with donor templates, large edits (268).
Base editors (CBE/ABE)	Precise single-base conversion without DSB; high efficiency within window	Editing window and bystander edits; sequence context; deaminase off-targets (DNA/RNA)	Repair of point mutations (transition edits), disease modeling (377).
Prime editors	Versatile (all base changes, small indels) without DSBs or donor	Variable locus efficiency; larger proteins/RNA components; delivery complexity	Precise “search-and-replace” edits where HDR is inefficient (378).
RNA editors (Cas13, other RNA effectors)	Transient, reversible modulation; antiviral diagnostics/therapies	Potential collateral activity; transient effect (needs repeat dosing); delivery to cytosol	Transcript knockdown, RNA base editing, antiviral applications, diagnostics (61).

CRISPR, clustered regularly interspaced short palindromic repeats; Cas, CRISPR-associated protein; DSB, double-strand break; HDR, homology-directed repair; PAM, protospacer adjacent motif; CBE, cytosine base editor; ABE, adenine base editor; RNA, ribonucleic acid.

Beyond Cas9, other CRISPR-associated proteins have significantly expanded the potential application. Cas12 (Type V) effectors differ from Cas9 by generating staggered double-strand breaks and exhibiting collateral ssDNA degradation, making them useful for genome editing and diagnostic applications such as DNA endonuclease-targeted CRISPR trans reporter (DETECTR) (62, 63). Cas14/Cas12f and engineered miniaturized variants such as miniaturized CRISPR-associated nuclease (CasMINI) provide compact alternatives suitable for delivery via adeno-associated viruses (AAVs), facilitating *in vivo* applications where vector size is limiting (64, 65). Cas13 (Type VI) targets RNA rather than DNA, and has been adapted into programmable RNA-editing systems such as RNA editing for programmable A to I replacement (REPAIR) and RNA editing for specific C to U exchange (RESCUE), which catalyze adenosine-to-inosine or cytosine-to-uracil conversions in transcripts, allowing transient, reversible editing without altering the genome (61, 66).

Emerging CRISPR-associated transposase (CAST) systems integrate RNA-guided targeting with transposon machinery, enabling programmable, DSB-free DNA insertions that bypass host repair pathways, thus increasing precision and efficiency for certain genome engineering applications (67).

Finally, CRISPR has been harnessed for gene regulation without altering DNA sequences. CRISPRa employ dCas9 fused to transcriptional activators to upregulate gene expression, while CRISPRi use dCas9-repressor fusions to silence gene transcription, facilitating functional genomics studies and potential therapeutic gene modulation (68, 69). Collectively, these platforms demonstrate the increasing diversification of CRISPR-based technologies, spanning DNA and RNA editing, precision base modifications, delivery system optimization, and transcriptional regulation, thereby expanding their translational potential in next-generation therapeutics.

## 3 Application of CRISPR–Cas in neurodegenerative disorders

### 3.1 CRISPR–Cas in Alzheimer's disease

AD is a progressive ND that impairs cognitive functions, including memory, reasoning, and daily living tasks (70, 71). The disease is a major cause of dementia among the elderly. It is characterized by the presence of amyloid-beta (Aβ) plaques and neurofibrillary tangles in the brain, which impair neurons and contribute to the degeneration of brain tissue (72, 73). The disease initiates with slight memory challenges that progress to severe cognitive and physical impairments, ultimately resulting in complete dependency and premature death (74). The disease disseminates throughout diverse communities at a prominent global health scale due to its elevated prevalence rates. There are 5.5 million individuals in the United States with AD. In 2016, the global number of individuals with dementia reached 43.8 million, reflecting a 116% increase since 1990, according to recent studies (75).

Development of AD largely driven by hereditary elements follows several pathophysiological paths. Many times, mutations in the *APP, PSEN1*, and *PSEN2* genes link fAD to one another (76). The *APP* gene codes the amyloid precursor protein on chromosome 21, which β-secretase and γ-secretase break to generate Aβ peptides. Applying mutations in *APP* produces overly high synthesis of Aβ, particularly the Aβ42 variant, which readily accumulates to generate amyloid plaques, a key hallmark of AD (77). Importantly, members of the γ-secretase complex, *PSEN1* and *PSEN2*, respectively encode PSEN1 and PSEN2 proteins (18, 78). Early-onset fAD is caused by mutations in these genes altering γ-secretase activity, hence increasing the Aβ42/Aβ40 ratio and promoting Aβ aggregation (79).

Apart from amyloid pathology, tau protein dysfunction also speeds the development of disease. Although *microtubule-associated protein tau* (*MAPT)* gene mutations are rare in AD, tau proteins in affected individuals hyperphosphorylated, therefore losing their ability to stabilize microtubules (80). Along with impairments in neuronal activity, this instability results in paired helical filaments and neurofibrillary tangles (81–83).

Moreover, exacerbating AD development is neuroinflammation. Released by active microglia and astrocytes, reactive oxygen species and pro-inflammatory cytokines damage neurons. Variations in the *triggering receptor expressed on myeloid cells 2* (*TREM2)* gene, which affects microglial activity, have also been associated to increased AD risk, therefore underlining the critical role immune responses play in the evolution of disorders (84–87).

**Figure 2:**
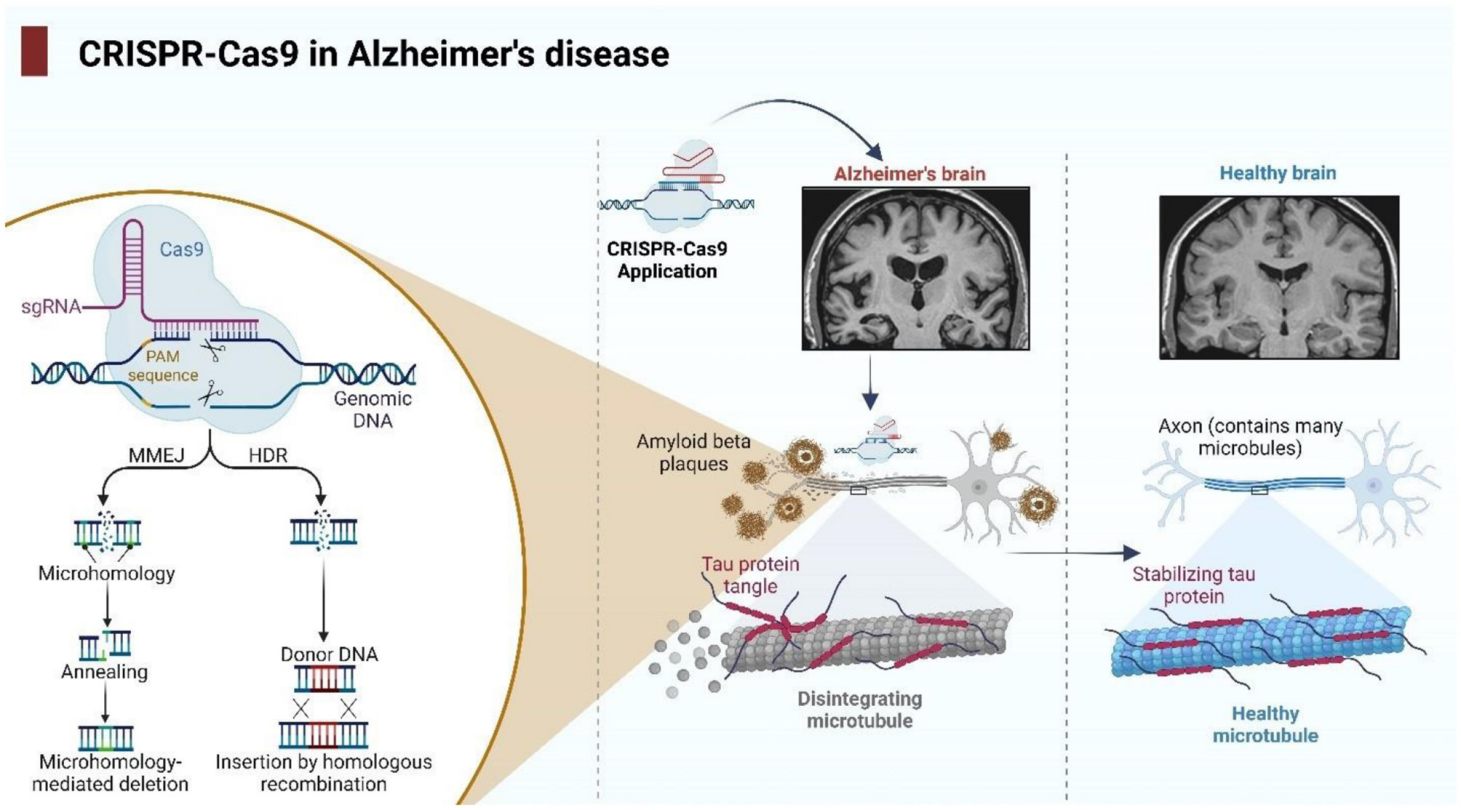
Diagrammatic illustration of the CRISPR–Cas9 system in the study and treatment of Alzheimer's disease (AD) and shows how CRISPR–Cas9 can be used to target genetic alterations and pathways linked to AD. The figure was designed with BioRender (http://Biorender.com/). CRISPR, clustered regularly interspaced short palindromic repeats; Cas9, CRISPR-associated protein 9; sgRNA, single-guide RNA; PAM, protospacer adjacent motif; HDR, homology-directed repair; MMEJ, microhomology-mediated end joining; DNA, deoxyribonucleic acid; Aβ (amyloid beta), amyloid-β peptide.

CRISPR–Cas9 represents a formidable gene-editing technique for AD, facilitating the precise modification of pathogenic genes, such as rectifying or eliminating *APP*, β*-site amyloid precursor protein cleaving enzyme 1* (*BACE1)*, and *PSEN1/2* mutations to diminish the production and aggregation of harmful Aβ (88); the conversion of Apo-lipoprotein E ε4 allele (*APOE4)* to the protective *APOE3 or APOE2* isoforms, thereby reducing Aβ accumulation, tau pathology, and neuronal susceptibility; the modulation of neuroinflammation through the targeting of CD33, glia maturation factor (GMF), or other immune mediators to enhance microglial clearance of amyloid plaques and inhibit detrimental cytokine signaling (89); and the amplification of neuroprotective pathways by promoting non-amyloidogenic APP processing (α-cleavage) or bolstering microglial functions that maintain neuronal integrity (88). These attributes render CRISPR a versatile method for rectifying genetic disease etiologies, reducing the accumulation of deleterious proteins, mitigating inflammation, and enhancing cerebral resilience. This approach enables researchers to acquire accurate AD models *in vivo* and *in vitro*, enhancing the comprehension of disease mechanisms, and aiding in the identification of novel therapy alternatives (Figure 2).

Cell models are extensively utilized in application on many neurodegenerative disorders, including AD, due to the absence of ethical dilemmas, the brevity of the experimental duration, and the cheap associated costs. Over the past few decades, numerous *in vitro* cellular models of AD have been developed.

Researchers have effectively employed CRISPR–Cas9 to eliminate the APP and *BACE1* genes in neural cell types. This resulted in a significant reduction in the synthesis of Aβ, a primary therapeutic target for AD. The previous study demonstrated that the ablation of APP in brain cells via CRISPR–Cas9 diminished both Aβ expression and amyloid pathology. This indicates its potential as a gene therapy alternative for individuals with APP mutations (89, 90). Sun et al. (91) employed CRISPR–Cas9 to modify the C-terminus of endogenous APP, markedly diminishing β-secretase cleavage and redirecting processing toward non-amyloidogenic α-secretase, thus decreasing Aβ generation.

Previous studies were conducted to reduce the Aβ utilizing CRISPR technique the knockout of *PSEN1 and PSEN2* genes (92–95). In addition, researchers effectively introduced this mutation into brain cells utilizing Cas9 nickase–deaminase, significantly reducing Aβ generation (96). Simultaneously, Song et al. (97) discovered that the suppression of KIBRA (Kidney and Brain expressed protein) in HT22 cells inhibited proliferation and induced death, whereas treatment with Aβ1–42 oligomers was administered (96, 98). Prime-Base editing was used to treat AD via utilizing CRISPR to correct the gene defects, and the results shows reduction in Aβ peptide accumulation. Several studies have been performed *in vitro* utilizing CRISPR–Cas9 technology to modify and editing the *APOE* gene, as this method has the potential to convert high-risk *APOE4* alleles into protective alleles such as *APOE3 or APOE2* to reduce the AD risk (99).

In addition to constructing AD cell models, CRISPR–Cas9 technology can also be utilized to develop AD animal models to improve and correct the cognitive function in AD. Serneels, T'Syen (100) Developed a novel model utilizing the CRISPR–Cas9 technique to construct a humanized A sequence in the *APP* gene of both species (100). Another study utilized animal models *in vivo* to improve cognitive function and correct *APP* and *BACE1* genes in AD. It demonstrated that the CRISPR system can suppress Aβ, having a significant effect on both pathology and cognitive function (101–103).

Furthermore, CRISPR-based gene editing techniques, particularly base editing and prime editing, exhibit the capacity to rectify harmful AD mutations without inducing double-strand DNA breaks. The genetic modification executed by base editors led to significant enhancements in tau pathology and cognitive recovery in the examined mice (58, 104, 105). *APOE* possesses various variations, including APOE2, APOE3, APOE3r, and APOE4. Komor et al. (59), the transfer of APOE3 to APOE4 in mouse astrocytes was achieved by changing Cincodon158 to T using the CRISPR–Cas9 system, indicating that point mutations were corrected by the CRISPR–Cas9 approach (59).

Prime-Base editing serves as a genome-editing technique that offers extensive genetic modification possibilities via mutation repair without inducing DSBs to address AD. *In vivo* research focused on AD-related applications needs greater exploration (104–106), such as a study by Park et al. (103) via utilizing Cas9 nanocomplexes with sgRNAs targeting the *tyrosine hydroxylase* and *BACE1* genes were administered into mouse primary neural cells to evaluate the efficacy of Cas9 nanocomplexes. Furthermore, Takalo et al. (107) utilizing the CRISPR–Cas9 gene editing technique and evaluating the preventive effects of the Plc2-P522R variant.

Patient-derived induced pluripotent stem cells (iPSCs) have garnered growing interest due to their distinctive physiological characteristics. AD mutant genes, such as *PSEN1, PSEN2*, and *APP*, in iPSC-derived neurons provide exceptional platforms for elucidating disease mechanisms and formulating novel therapeutic strategies. Studies demonstrated that gene editing using CRISPR–Cas9 can mitigate the impairments linked to AD by addressing synaptic dysfunction and altered signaling pathways (108–111). Researchers have created gene-edited induced pluripotent stem cell models to investigate genetic mutations associated with AD, as well as knockout genes implicated in Alzheimer's pathogenesis, including ATP-binding cassette subfamily A member 7 (ABCA7) (112–114). The integration of animal models and CRISPR-edited iPSCs enhances scientists' understanding of AD risk factors and therapeutic options (115). CRISPR-engineered iPSC and organoid models have elucidated critical mechanisms in AD, including lipid metabolism (*APOE*), endosomal dysfunction (*APP/PSEN1*), electrophysiological abnormalities (*PSEN2*), and neuroimmune interactions (*APOE Christchurch*). These human-relevant systems are highly effective for identifying disease pathways and evaluating treatment alternatives (116–119). Stem cell models integrated with gene editing technologies facilitate the development of patient-specific therapies, paving the way for clinical applications.

Stem cell-derived models, in conjunction with gene editing, exhibit potential to enhance the development of tailored AD therapies. Table 2 Illiterates applications of CRISPR–Cas in AD. Utilizing CRISPR, scientists acquire enhanced insights into AD progression and develop successful therapeutic approaches.

**Table 2:** Applications of CRISPR–Cas in Alzheimer's disease.

**Gene target**	**Model**	**Editing modality**	**Delivery vector/method**	**Result**	**Refs**.
*BACE1* (reduce Aβ production)	*In vivo* mouse AD models	CRISPR-Cas9 nuclease (knockdown/KO)	Nanocomplexes/AAV or RNP nanocarriers (in-brain delivery)	Targeting Bace1 in adult mouse brain reduced Aβ pathology and improved cognitive readouts in two AD mouse models. Demonstrates feasibility of in-brain CRISPR editing to lower amyloid.	(103)
*APP* (protective A673T/pathogenic mutations)	*In vitro* (neurons, iPSC) & *in vivo* ambitions	Base editing and prime editing to install/correct single-base changes (A673T protective variant; familial *APP* mutations)	Plasmid/BE mRNA transfection or AAV/dual-AAV for *in vivo* prime editing	Base editors and prime editors have been used to insert the protective A673T (or correct pathogenic *APP* alleles) in cells and tested for translation to neurons/mouse models showing precise installation with lower indels vs. nuclease.	(96)
*APOE4 → APOE3* correction/modeling	Human iPSC lines → neurons/ astrocytes; organoids	CRISPR-Cas9 HDR (isogenic lines), prime editing (optimized pegRNAs)	Electroporation/RNP/plasmid transfection for iPSC; dual-AAV prime editing for *in vivo* approaches	Isogenic CRISPR correction of *APOE4 → APOE3* in human iPSC altered lipid metabolism and neuronal/astrocyte phenotypes; optimized prime editing pipelines enable high-efficiency correction in iPSCs/organoids.	(379)
*TREM2* (microglial function modulation)	*In vitro* microglial lines (BV2), *in vivo* mouse models	CRISPR-Cas9 knockout/knock-in; gene dosage modulation (transgene)	Lentiviral/CRISPR plasmid transfection for cell lines; genetic transgenesis or viral approaches in mice	CRISPR-engineered *TREM2* variants in microglia change phagocytosis and inflammatory signatures; increasing *TREM2* dosage reprograms microglia and ameliorates neuropathology in AD models.	(380)
*MAPT* (tau)/tauopathy modeling or modulation	iPSC neurons, organoids, transgenic mice	CRISPR-Cas9 (mutant introduction or correction), CRISPRi for expression modulation	Plasmid/viral vectors (lentivirus/AAV) *in vivo* or *in vitro* transfection	CRISPR has been used to introduce tau mutations into iPSCs/organoids to model tau pathology and to test tau-lowering strategies; CRISPRi approaches can reduce MAPT expression in neuronal models.	(381)
*Sortilin-related receptor 1 (SORL1), PSEN1/PSEN2* (fAD genes)	iPSC & organoid models; some mouse models	CRISPR-Cas9 HDR (knock-in/knock-out); base/prime editing for single-base changes	Electroporation/RNP/lentivirus for cellular work; transgenic approaches in animals	Generation of isogenic iPSC and organoid models carrying *PSEN1/2* or *SORL1* variants demonstrates altered Aβ processing and cellular phenotypes useful for mechanistic studies and preclinical testing.	(382)
Protective mutation installation *in vivo* (proof-of-concept)	Mouse brain (astrocytes & neurons)	Prime editing (dual-AAV PE systems)	Dual-AAV pegRNA + PE machinery (v3em PE-AAV systems)	*In vivo* prime editing in mouse brain achieved therapeutically relevant editing efficiencies (reported up to ~40% in cortex) and installed putative protective mutations for AD in astrocytes with minimal off-target readouts.	(383)
Organoids: multi-cell modeling (amyloid, tau, microglia interactions)	3D cerebral organoids from CRISPR-edited iPSCs	CRISPR-Cas9 editing in iPSC stage (HDR, base/prime editing) → differentiate into organoids	Electroporation/transfection at iPSC stage; viral transduction of progenitors	CRISPR-edited iPSCs permit building organoids that recapitulate familial/sporadic AD features (plaque-like amyloid, tau changes, altered microglia responses), enabling mechanistic and drug-testing platforms.	(384)

AD, Alzheimer's disease; CRISPR, clustered regularly interspaced short palindromic repeats; Cas9, CRISPR-associated protein 9; Aβ, amyloid-β peptide; iPSC, induced pluripotent stem cell; KO, knockout; HDR, homology-directed repair; RNP, ribonucleoprotein complex; AAV, adeno-associated virus; BE, base editing/base editor; PE, prime editing; pegRNA, prime-editing guide RNA; APP, amyloid precursor protein; BACE1, β-site amyloid precursor protein cleaving enzyme 1; APOE, apolipoprotein E; TREM2, triggering receptor expressed on myeloid cells 2; MAPT, microtubule-associated protein tau gene; PSEN1/PSEN2, presenilin 1 and 2; SORL1, sortilin-related receptor 1; 2D, two-dimensional cell culture; 3D, three-dimensional culture.

Jia et al. (120) created a CRISPR-powered aptasensor that detects Aβ biomarkers via fluorescence detection via CRISPR-Cas12a. There is currently little study on the use of CRISPR-Cas13 and CRISPR-Cas3 in AD diagnosis. Although its potential in diagnostic domains has not been thoroughly investigated, Ca3′s primary DNA deletion function remains its most prominent feature.

### 3.2 CRISPR/Cas in Parkinson's disease

PD, the second prevalent neurodegenerative ailment, is marked by the degradation of dopaminergic neurons, which results in particular motor symptoms such as rigidity, bradykinesia, and tremor (121). After the loss of neuronal, the Lewy bodies appeared with cytoplasmic inclusions, which are mostly composed of aggregates of α-synuclein protein misfolded and perhaps diffusion in a prion-like shape among interconnected areas synoptically (122). PD can be classified into two forms: sporadic and familial. The familial form accounts for only 10%−15% of all PD cases. The hereditary versions are attributed to mutations in multiple genes, including *SNCA, parkin (PRKN), PTEN-induced kinase 1 (PINK1), DJ-1/Parkinson's disease protein 7 (PARK7), and LRRK2. SNCA* and *LRRK2* mutations cause autosomal-dominant types of PD, while *PRKN, PINK1*, and *DJ-1* mutations cause autosomal recessive variants (123). Although the origins of sporadic forms are currently unknown, certain genetic and environmental risk factors have been discovered. For instance, genetic polymorphisms in the *LRRK2* and *SNCA* genes elevate the likelihood of developing sporadic PD (124).

Between 1992 and 2021, global PD cases escalated from 450,000 to 1.34 million, with crude incidence rates increasing from 8.19 to 16.92 per 100,000 and age-standardized incidence rates rising from 11.54 to 15.63 per 100,000. Incidence rates increased significantly in persons aged 60 and beyond, reaching a zenith in those aged 85 and above. Forecasts indicate that by 2030, worldwide PD cases will total 1.93 million, with an age-standardized incidence rate of 27 per 100,000 (125).

PD is a ND mostly attributed to genetic abnormalities and environmental stresses (126). Genetic alterations in critical genes such as *SNCA, LRRK2, PARK2, PINK1*, and *Glucocerebrosidase 1 (GBA1)* are central to the disorder. Mutations in *SNCA* result in the improper folding of SNCA protein, leading to aggregation into Lewy bodies, which are indicative of PD in the brain (127). Mutations in *LRRK2* that enhance gene function result in increased kinase activity, which is detrimental to neurons. Mutations in *PARK2* and *PINK1* disrupt mitochondrial quality control, leading to mitochondrial malfunction, poor mitophagy, oxidative stress, and ultimately, the demise of dopaminergic neurons (128). Neurons exhibit increased toxicity when mutations in the *LRRK2* gene lead to the enzyme acquiring excessive kinase activity. The *PARK2* gene regulates parkin activity, which acts as an E3 ubiquitin ligase; however, its dysfunction results in the buildup of protein damage (128). Neuronal damage exacerbates when *GBA1* mutations induce mitochondrial malfunction and elevate oxidative stress inside cells (129, 130). Genetic abnormalities alter protein degradation pathways, impair mitochondrial function, and interfere with oxidative stress responses, ultimately leading to PD symptoms through the degeneration of dopaminergic neurons in the substantia nigra (131). Impaired mitochondria result in heightened oxidative stress and diminished ATP synthesis, causing neuronal damage. The development of PD occurs due to mutations in *PINK1* and *PRKN* that impair mitochondrial quality control mechanisms (131). PD neuroinflammation occurs when activated microglia secrete tumor necrosis factor-alpha (TNF-α), interleukin (IL)-1β, and reactive oxygen species, which concurrently harm dopaminergic neurons. The stimulation of microglial cells by misfolded SNCA protein initiates a persistent inflammatory response. Extracellular immune cells, in conjunction with activated astrocytes, facilitate the progression of PD (132).

CRISPR–Cas techniques have proven essential in elucidating PD pathophysiology by facilitating the precise alteration of genes associated with dopaminergic neuron susceptibility (133). CRISPR-mediated knock-in and knock-out mice of *LRRK2, PINK1*, and *PARK* have clarified abnormalities in mitochondrial quality control and dysregulation of autophagy (134, 135). CRISPR correction of *LRRK2-G2019S* in patient-derived iPSCs reinstates dopaminergic differentiation and mitochondrial function, underscoring its therapeutic significance. CRISPRi has been utilized to diminish *SNCA* overexpression, hence decreasing aggregation and related toxicity (136). Novel therapeutic approaches encompass AAV-mediated CRISPR–Cas systems aimed at SNCA regulatory elements and base editors intended to rectify pathogenic point mutations; nonetheless, challenges related to delivery efficiency and off-target effects persist as significant obstacles (134).

CRISPR–Cas9 technologies, encompassing knockout, base editing, and gene correction, facilitate our comprehension of biological mechanisms and preserve phenotypes in models pertinent to PD. They possess significant potential for future gene therapy as they target genetic factors such as *LRRK2, PINK1*, and *PARKIN*, thereby aiding in the reduction of neuroinflammation and neuronal degeneration (137–140).

Recently, CRISPR–Cas9 genome editing has been employed in a laboratory to rectify mutations responsible for PD and to elucidate their pathogenic mechanisms. For example, employing CRISPR–Cas9 to eliminate the A53T-SNCA mutation resulted in a significant reduction in both *SNCA* mRNA expression and its protein levels (137, 141). Beligni et al. (142) demonstrated that CRISPR–Cas9 could rectify the LRRK2 p.Gly2019Ser (G2019S) mutation in patient-derived cells. This indicates its potential as a therapeutic agent to restore normal kinase function (143). A separate work employed CRISPR–Cas9 to entirely eliminate PINK1 in THP-1 cells, enhancing our understanding of PIN1′s function in maintaining mitochondrial integrity and the progression of PD (142). A recent work applied by (142) addresses the role of *PINK1* in PD by employing CRISPR–Cas9 to generate *PINK1* knock-out THP-1 cells. The *PINK1* knockout exhibited diminished mitochondrial complex I activity alongside alterations in Tau protein expression patterns. In *PINK1* knock-out macrophages, pro-inflammatory cytokines such as IL-6, IL-1β, and IL-23 were raised, while the lipid droplet count per cell decreased, indicating mitochondrial dysfunction and immune system pathological alterations (142).

Tokuda et al. expanded upon these findings by employing HDR to correct LRRK2 G2019S and truncate the LRRK2 kinase domain in marmoset embryonic stem cells and iPSCs. This rendered non-human primate cellular models applicable (144). In pertinent research, elevating glial cell line-derived neurotrophic factor (GDNF) levels in MPTP-treated neuronal cells via CRISPR–Cas9 was associated with reduced monoamine oxidase B (MAO-B) activity, diminished production of reactive oxygen species, and enhanced cell survival under neurotoxic stress (145). The *in vitro* applications of CRISPR–Cas9, including gene deletion, point mutation correction, gene knockout, and gene upregulation, demonstrate its versatility and efficacy as a tool for investigating the biology of PD and developing gene-based therapies.

Researchers have employed CRISPR–Cas9 to develop large-animal models (*in vivo*) of PD by introducing deleterious *SNCA* mutations into existing brain systems (146). Zhu et al. (146) employed CRISPR–Cas9 and *SCNT* to generate Guangxi Bama minipigs with the PD-associated E46K, H50Q, and G51D mutations in *SNCA*. Despite the presence of mutant *SNCA* in the early animals, they exhibited neither dopaminergic neurodegeneration nor Lewy body pathology at 3 months, highlighting the potential and limitations of this large-animal paradigm. Employing dCas9, CRISPRi effectively reduced SNCA mRNA and its protein levels in animal models (147). *PINK1* and *PARKIN* constitute a pivotal signaling axis that significantly regulates the mitochondrial autophagy process in dopaminergic neurons. In *PINK1* null Drosophila, the silencing of the *ubiquitin c-terminal hydrolase L1* (*UCHL1)* gene using RNA interference can ameliorate the pathology associated with PD. The absence of UCH deubiquitination enhances mitochondrial autophagy by stimulating the expression of *AMP-activated protein kinase (AMPK) and Unc-51–like autophagy-activating kinase 1 (ULK1)* (148).

Researchers have developed the mitochondrial complex I (MCI)-Park model in mice using CRISPR Cas9 to generate dopamine neurons deficient in NADH dehydrogenase [ubiquinone] iron–sulfur protein 2 (NDUSF2), which encodes mitochondrial complex I. Mice deficient in NDUSF2 exhibited neurodegenerative alterations (149). Point mutations in the vacuolar protein sorting 35 gene (VPS35) are linked to autosomal dominant late-onset PD (PARK17). The homozygous deletion of Vps35 using CRISPR Cas9 led to a survival disadvantage and a marked decrease in dopamine release in the caudate putamen of adult homozygous Vps35 mutant mice (150). Peroxidized phospholipids resulting from ferroptosis have been linked to the beginning of certain PD. Patatin-like phospholipase domain-containing protein 9 (PNPLA9), functioning as a hydrolytic enzyme, exhibits a preference for hydrolyzing peroxidized phospholipids. In mice, the ablation of PNPLA9 led to the advancement of Parkinsonian motor abnormalities and the accumulation of peroxidized phospholipids (151). This represents a promising method for gene repression *in vivo* PD models.

Researchers have investigated the application of CRISPR techniques to activate genes. Narváez-Pérez et al. employed a pseudo-lentivirus to deliver a CRISPR/SAM system to rat astrocytes, enhancing the expression of tyrosine hydroxylase and resulting in dopamine production. When these altered astrocytes were introduced into a rat model exhibiting 6-hydroxydopamine (6-OHDA)-induced injury, they improved motor function (138). This demonstrated an innovative approach to treating PD. Increased CRISPR-Cas9 genome editing has produced mouse models that eliminate NDUSF2 (mitochondrial complex I) or knock out VPS35 and Pnpla9 (148–152). This induces neurodegeneration and motor dysfunction akin to the manifestations of PD. Conversely, the ablation of *PRKN, PINK1*, and *DJ-1* thrice in murine models did not induce neurodegeneration, indicating that some species exhibit greater resilience and that the genetic modeling of PD is complex (153).

iPSC models derived from individuals with PD are valuable for investigating genetic variations associated with the disorder, such as *LRRK2, SNCA, PARKIN*, and *ubiquinol–cytochrome c reductase core protein 1* (*UQCRC1)* (154–156). Neural stem cells (NSCs) harboring the *LRRK2* p.G2019S mutation are predisposed to proteasome stress, nuclear envelope dysfunction, clonal proliferation issues, and challenges in neural differentiation (157).

Moreover, rectifying this mutation using knock-in techniques restores the phenotype to normal, indicating that nuclear morphology may serve as a biomarker for PD. LRRK2 p.G2019S disrupts mitochondrial mitophagy by impairing the Miro–PINK1/PARKIN pathway, hence hindering cellular respiration and metabolism (158). Similarly, NSCs derived from individuals with *PARK2* mutations differentiate into neurons exhibiting elevated oxidative stress, activated Nrf2 pathways, and abnormal mitochondrial morphology and dysregulated homeostasis (159, 160).

Research on dopaminergic SH-SY5Y cells with *UQCRC1* mutations, observed in both familial and sporadic PD, indicates that axonal degradation occurs and mitochondrial respiratory chain function ceases. These iPSC-derived cellular models demonstrate how genetic risk factors influence pathways governing proteostasis, mitochondrial integrity, oxidative equilibrium, and cellular morphology (161). They demonstrate the utility of CRISPR–Cas9-mediated gene repair or knockout in restoring cellular function, thereby enhancing the understanding of underlying mechanisms and identifying potential therapeutic targets in PD.

CRISPR-modified PD organoids replicate dopaminergic neuron degeneration, oxidative stress, and α-synuclein pathology, offering a three-dimensional framework for longitudinal investigations. Three-dimensional human midbrain organoids generated from CRISPR-modified induced pluripotent stem cells augment the physiological relevance of these models. These organoids have a spatial organization akin to the human substantia nigra, with dopamine-producing neurons, astrocytes, and nascent microglial-like populations. CRISPR-induced PD mutations in organoids expedite SNCA fibrillization, impair neuromelanin production, cause lysosomal dysfunction, and lead to progressive neuronal loss, reflecting clinical illness with temporal advancement. These 3D systems enable longitudinal examination of synapse degeneration, neuroinflammation, and intercellular communication processes inadequately represented in 2D cultures (162).

Biomedical applications illustrate the usefulness of CRISPR-Cas12 technology, which has shown promise in research for treating NDs, including PD. Healthcare research continues to develop and still under research, studies on the applicability of CRISPR-Cas12 for PD treatment, utilizing the existing CRISPR technology still limited.

Researchers have discovered intriguing applications for CRISPR-Cas3′s RNA-targeting DNA editing technologies in treating NDs, including PD. CRISPR systems based on DNA editing fail to target single-stranded RNA, but Cas13 can precisely control gene expression using RNA targets that do not alter genomic sequences (163). The application on PD and CRISPR-Cas13 still limited and shown in Table 3.

**Table 3:** Applications of CRISPR–Cas in Parkinson's disease.

**Gene target**	**Model**	**Editing modality**	**Delivery vector/method**	**outcomes**	**Refs**.
*SNCA* (α-synuclein) — lower expression or correct copy number	*In vitro*: iPSC-derived neurons (*SNCA* triplication) and neuronal cultures. *In vivo*: rodent brains (proof-of-concept). Organoids: midbrain organoids with SNCA triplication.	CRISPR-Cas9 nuclease (KO), CRISPRi (dCas9 repression), CRISPR editing to model triplication.	*In vitro*: electroporation/ plasmid, lentivirus, RNP delivery. *In vivo*: AAV/brain injections in rodents (experimental).	CRISPRi or targeted editing reduces *SNCA* expression and mitigates oxidative stress/mitochondrial damage in iPSC-derived neurons; SNCA triplication organoids recapitulate α-syn pathology and are useful for testing interventions. *In vivo* knockout proof-of-concept shows phenotype modulation in animal models.	(242)
*LRRK2* (e.g., G2019S) — disease modeling and mutation correction	*In vitro*: iPSC lines → dopaminergic neurons; some mouse transgenic models for phenotyping.	CRISPR-Cas9 HDR (knock-in/correction), double-nicking, base editor applications reported in reviews; prime editing examined in methods literature.	*In vitro*: electroporation/RNP/plasmid + HDR templates. For potential *in vivo* translation: viral strategies (AAV/HDAdV discussed in reviews).	CRISPR correction of *LRRK2* G2019S in patient iPSC restores cellular phenotypes (reduced ROS, normalized Neurite morphology and restored autophagy deficiencies; many groups have produced isogenic strains for mechanistic investigations). Base and prime editors are presented as safer alternatives for the repair of point mutations.	(144)
*GBA* (glucocerebrosidase) model & functional studies	Human iPSCs from PD patients; isogenic controls via CRISPR → differentiated into neurons/organoids.	CRISPR-Cas9 HDR (knock-in/ correction) to make isogenic lines; CRISPR used to model *GBA* variants.	Electroporation/RNP/ plasmid transfection in iPSCs; lentiviral approaches for some assays.	CRISPR-generated isogenic iPSC lines with *GBA* mutations replicate diminished GCase activity and elevated α-synuclein accumulation; corrected lines ameliorate biochemical and behavioral abnormalities, providing a robust platform for mechanistic studies and pharmacological testing.	(385)
*PRKN (PARKIN) & PINK1* mitophagy pathways/loss-of-function models	iPSC-derived dopaminergic neurons, organoids, and cellular lines; some mouse KO models used to study pathway effects.	CRISPR-Cas9 KO/knock-in of disease variants; prime editing proposed for point mutations.	Electroporation/lentiviral transduction/RNPs for cells; genetically engineered mouse lines for *in vivo* studies.	CRISPR knockout or mutation induction results in mitophagy and mitochondrial dysfunction phenotypes in neurons and organoids; these models are utilized to investigate rescue techniques and evaluate small drugs. Prime editing is noted for its capacity for exact edits; yet, there are currently few reported examples of in-brain PD applications.	(386)
Therapeutic delivery & editing modalities (base/prime) — proof-of-concept & methods	*In vivo* brain editing (rodent) and methods papers; *in vitro* correction in iPSCs and neurons.	Base editors (ABE/CBE) applied to point mutations in other neuro genes; prime editing proposed/demonstrated in rodent brain in general (methods).	Dual-AAV prime editor systems (method papers), non-viral nanoparticles and RNPs discussed for safer delivery.	Method studies illustrate the transport of primary editing to brain tissues and examine its application to PD-related genes (*SNCA, LRRK2, PRKN)* as potential future treatments; base editors are utilized *in vitro* to create or rectify point mutations with a reduced incidence of double-strand breaks.	(387)
CRISPRi/epigenetic targeting of *SNCA* promoter or intron 1	iPSC-derived neuronal cultures from *SNCA* triplication patients (*in vitro*)	CRISPRi (dCas9-KRAB) or targeted epigenetic editing (methylation)	Lentivira, plasmid transfection, dCas9 fusion proteins delivered *in vitro*	CRISPRi directed at the *SNCA* promoter/intron diminishes α-syn expression, alleviates oxidative stress, and mitigates mitochondrial DNA damage, providing a reversible, non-double-strand break method to reduce hazardous protein levels.	(388)

PD, Parkinson's disease; CRISPR, clustered regularly interspaced short palindromic repeats; Cas9, CRISPR-associated protein 9; iPSC, induced pluripotent stem cell; KO, knockout; HDR, homology-directed repair; CRISPRi, CRISPR interference; dCas9, catalytically inactive Cas9; KRAB, Krüppel-associated box transcriptional repression domain; ABE, adenine base editor; CBE, cytosine base editor; RNP, ribonucleoprotein complex; AAV, adeno-associated virus; HDAdV, helper-dependent adenoviral vector; ROS, reactive oxygen species; SNCA, α-synuclein gene; LRRK2, leucine-rich repeat kinase 2; GBA, glucocerebrosidase gene; PRKN (PARKIN), parkin gene; PINK1, PTEN-induced kinase 1; mtDNA, mitochondrial DNA.

### 3.3 CRISPR-Cas in Huntington's disease

HD is a progressive ND, an autosomal dominant disease. The condition is attributed to an enlarged cytosine-guanine-adenine (CAG) repeat in the *HTT* gene located on chromosome 4 (4p16.3), resulting in a *mutant huntingtin (mHTT)* protein characterized by an elongated polyglutamine tract (164–166). The deleterious gain-of-function characteristics of mHTT initiate a series of molecular disruptions that ultimately result in the specific destruction of medium spiny neurons within the striatum. At the molecular level, mHTT impairs transcriptional control by anomalously interacting with transcription factors, including CREB-binding protein (CBP), resulting in extensive transcriptional suppression and modified neuronal identity. Moreover, mHTT disrupts axonal transport by obstructing microtubule-associated motor proteins (dynein/dynactin), leading to impaired trafficking of vesicles, mitochondria, and neurotrophic signals such as brain-derived neurotrophic factor (BDNF) (167). Individuals with optimal health typically possess 16–20 CAG repeats, however, those afflicted with HD may have over 40 repetitions. Extended repeats are associated with earlier onset and more severe symptoms (164). The misfolded mHTT protein forms toxic aggregates that disrupt critical cellular activities such as transcription, energy metabolism, axonal transport, and apoptosis. This ultimately results in the demise of striatal and cortical neurons. In the clinic, HD manifests in middle age with issues related to movement, cognition, and mental health (168). The studies detected the mitochondrial dysfunction is a primary characteristic, marked by diminished respiratory chain activity, compromised calcium buffering, elevated oxidative stress, and increased vulnerability to apoptosis. mHTT also undermines autophagy and proteostasis by obstructing autophagosome cargo loading, impairing ubiquitin–proteasome functionality, and facilitating the formation of insoluble aggregates. Synaptic dysfunction manifests early via altered NMDA receptor activation, excitotoxicity, and compromised corticostriatal connection. Recent data indicates that non-cell-autonomous mechanisms, including as impaired astrocytic glutamate absorption and microglial activation, contribute to the acceleration of neuronal death. These interrelated pathways collectively characterize HD as a progressive multisystem condition influenced by proteotoxicity, transcriptional dysregulation, compromised energy metabolism, and synaptic susceptibility (169, 170). A 2012 meta-analysis revealed significant regional variation in the prevalence of HD, with elevated rates in Europe, North America, and Australia (5.7 per 100,000) compared to diminished rates in Asian populations (0.4 per 100,000) (171).

HD pathophysiology comes from the misfolding of mHTT into aggregates that impair cellular function, ultimately leading to neuronal cell death (172). The protein interferes with transcriptional regulators, leading to aberrant gene expression throughout the organism (173). Mitochondrial dysfunction, along with elevated oxidative stress, induces neuronal injury by diminishing cellular energy levels (174). Concurrent aberrant glutamate activity induces neuronal death pathways. The mHTT protein induces malfunction in the proteasome and autophagy processes, leading to the accumulation of toxic proteins (175). Neuronal damage increases due to chronic neuroinflammation caused by activated microglia and astrocytes in the nervous system (176). The prospective applications of CRISPR-Cas technology for the treatment of HD are significant due to its capacity for precise genetic material alteration. Gene therapy employing targeted CRISPR-Cas9 serves as a prospective treatment strategy for disease management by directly correcting genetic mutations at their origin (164, 177). Various studies have been demonstrated the potential of CRISR/Cas tool in HD application and theoretical aspects such as gene editing, selective targeting of mutant alleles, and excision of expanded cag repeats (178–180). Shin et al. undertook a study with the goal of enhancing the specificity of alleles. They employed a customized approach using CRISPR–Cas9 that selectively targeted specific alleles by modifying PAM-altering single nucleotide polymorphism (SNP). This approach focused on identifying CRISPR–Cas9 sites that were unique to each patient and utilized a comprehensive understanding of the haplotype structure of the *HTT* gene. The objective was to deactivate the mutated *HTT* allele for a specific haplotype specifically (181).

Researchers have employed CRISPR–Cas9 genome editing to create isogenic cell models (*in vitro*) of HD by precisely modifying the CAG repeat region in the *HTT* gene. CRISPR has been used to allele-specific targeting and genetic correction, Allele-specific excision of the mutant *HTT*, Shin employed SNP-guided dual-CRISPR–Cas9 targeting to excise approximately 44 kb from the *mHTT* allele in patient-derived cells. This eliminated mutant mRNA and protein while preserving the wild-type allele (181). In a separate investigation, Yang et al. showed that the application of CRISPR–Cas9 in HD140Q-KI mice to remove *HTT* in a non-allele-specific way can successfully and permanently eradicate the harmful effects of polyglutamine expansion on neurons in the adult brain. The study population showed a significant reduction in reactive astrocytes and a notable improvement in motor impairment (182). Another study utilized CRISPR to CAG-repeat excision, such as a study introduced 150 CAG repeats into exon 1 of HEK293T cells to create clones resembling the condition, perhaps facilitating the investigation of disease mechanisms (183). In pertinent research, scholars employed Cas9 ribonucleoprotein complexes and single-stranded oligodeoxynucleotide donors to create HEK293T lines containing 41, 53, and 84 CAG repeats. This facilitated the examination of alterations in cellular phenotypes associated with varying repeat lengths and contributed to the development of novel treatments (184). Another approach employed Cas9 nickases and guide RNAs to excise CAG repeats, resulting in frameshifts that activated premature stop codons and inhibited *mHTT* production. This illustrates how accurate genome editing could reinstate the typical allele configuration (185). In transgenic BacHD mice, Monteys et al. (186) incorporated a modified human HD allele and targeted human *m*HTT exon 1. BacHD animals treated with rAAV.spCas9 and rAAV.sgHD1B/i3 showed decreased HTT mRNA levels in their right hemisphere but not in their uninfected left (186). Ekman et al. (187) revealed effectively inhibited the expression of the human mutant *HTT* gene in R6/2 mice that carried human *mHTT* exon 1 with roughly 115–150 CAG repeats in 2019 using AAV-mediated CRISPR-Cas9. This study showed that R6/2 mice's lifespan and motor deficits were enhanced by inhibiting *HTT* expression. In conclusion, it has been shown in various animal models that HD may be effectively treated by employing CRISPR-Cas9 to eliminate *mHTT* gene expression (187).

CRISPR-based gene-silencing techniques have been employed to reduce the expression of *mHTT* with the direct deletion of repeat expansions. CRISPRi exclusively inhibited *mHTT* transcription in fibroblasts derived from HD patients, without affecting wild-type *HTT* (188). Previous work employed CasRx (RNA-targeting Cas13) to downregulate *HTT* exon 1 in HEK293T cells, HD 140Q knock-in mice, and HD knock-in pigs. This decreased mHTT levels, diminished gliosis, and decelerated neurodegeneration (189). Ekman et al. (187) revealed effectively inhibited the expression of the human *mHTT* gene in R6/2 mice that carried human *mHTT* exon 1 with roughly 115–150 CAG repeats in 2019 using AAV-mediated CRISPR-Cas9. This study showed that R6/2 mice's lifespan and motor deficits were enhanced by inhibiting *HTT* expression. It has been shown in various animal models that HD may be effectively treated by employing CRISPR-Cas9 to eliminate *mHTT* gene expression (187).

Oura et al. (190) addressed the target range limits of WT-SpCas9, scientists engineered SpCas9-NG, which detects NGN PAMs. They used SpCas9-NG to precisely contract HD CAG repeat tracts in mouse embryonic stem cells. The procedure of rectifying mutations restored distinctive characteristics as well as health conditions in both neurons derived from modified stem cells and the animals produced by these cells. This study reveals that SpCas9-NG is a powerful way to treating enlarged CAG repeats and other disease variants that are difficult to target (190). Another study via Merienne et al. (191) found the development of the KamiCas9 self-inactivating CRISPR–Cas9 system, which enables high-efficiency Cas9 expression during transitory intervals. Using their technique on adult mouse neuronal and glial cells, researchers were able to inactivate the *HTT* gene while concurrently improving HD disease markers. The KamiCas9 system made it possible to reduce unwanted edits when evaluating human iPSC-derived cells since it had less off-target effects than typical constitutive Cas9 systems. The study shows how self-inactivating CRISPR–Cas9 technologies could become a feasible therapy option for NDs (191). Ultimately, allele-specific CRISPR–Cas9 editing utilizing SNP-generated PAM sites facilitated the targeted silencing of *mHTT* by nonsense-mediated degradation in approximately 20% of European HD patients. This demonstrated exceptional accuracy and little off-target effects following GUIDE-Seq validation. This research demonstrates the versatility of CRISPR approaches in investigating the etiology of HD and formulating novel therapies. They can be utilized for genome editing, gene silencing, and allele-specific strategies (192).

CRISPR-based techniques have been shown to be effective *in vivo* for HD by reducing levels of mHTT in the brain. Bunting and Donaldson demonstrated that employing antisense oligonucleotides to inhibit MSH3 activity reduced HTT CAG repeat expansions in HD mouse models. This indicates that gene-targeting methodologies may alter the trajectory of the disease (193). Similarly, administering CRISPR–Cas9 to striatal neurons in HD140Q-knockin mice significantly reduced endogenous mHTT, inhibited aggregation formation, and ameliorated early symptoms of neurodegeneration and motor dysfunction, all without causing neuronal damage (182). In the prevalent R6/2 mouse model, CRISPR–Cas9-induced deletion of *HTT* resulted in reduced protein inclusions, enhanced motor function, and an extended longevity. This indicates that targeted genome editing may be an effective approach for treating HD (103, 186, 187, 191).

Enhanced editing precision has significantly improved safety and efficacy. Base editing was utilized *in vivo* to disrupt caspase-6 cleavage sites within the *HTT* gene. This resulted in *mHTT* isoforms that exhibit resistance to proteolytic processing. This approach effectively reduced the detrimental aggregation of huntingtin and safeguarded against neuronal loss in the striatum and brain of HD animal models (187). *In vivo* investigations, encompassing antisense, CRISPR–Cas9 gene disruption, and base editing, demonstrate that reducing or altering *mHTT* expression can decelerate the advancement of neuropathological disorders and enhance functional outcomes. This indicates that CRISPR possesses the potential to serve as a disease-modifying instrument for HD (194).

The integration of iPSCs with CRISPR–Cas technology has advanced HD research by creating precise, isogenic models and revitalizing cells. Scientists developed homozygous HEK293T cell lines with varying amounts of CAG repeats in the *HTT* gene to facilitate mechanistic investigations and drug screening (184). Researchers have employed CRISPR–Cas9 to rectify pathogenic CAG expansions in iPSCs, effectively reversing initial indicators of mitochondrial dysfunction in HD-derived neural stem cells, including respiratory issues, elevated reactive oxygen species, and calcium dysregulation (195–198). The results indicate that the selective elimination of CAG repeats can restore mitochondrial function and maintain cellular equilibrium. CRISPR–Cas9 editing facilitates allele-specific disruption, repeat excision, and gene repair in both *in vitro* and *in vivo* environments. Outstanding issues remain, including its efficacy, long-term safety, and performance on alternative genes. This gene-editing technique possesses significant potential to alter the trajectory of HD (199). Table 4 illustrates some HD applications and consequences.

**Table 4:** Applications of CRISPR–Cas in Huntington's disease.

**Gene target**	**Model**	**Editing modality**	**Delivery vector/method**	**Result**	**Refs**.
*HTT* allele-selective disruption (promoter/exon 1 deletions)	*In vitro*: patient fibroblasts & iPSC-derived neurons. *In vivo*: transgenic mice expressing human *mHTT*.	CRISPR–Cas9 nuclease (paired guides or single-site targeting for frameshift) allele-selective using PAM-creating/destroying SNPs	AAV for *in vivo* brain delivery; plasmid/RNP/lentivirus for cells.	Allele-selective targeting (utilizing SNPs that modify PAMs) or exon 1 deletions diminish mutant *HTT* expression, decrease aggregates, and improve some neuropathological features and phenotypes in murine models and patient-derived cells. Exhibits the viability of permanently inactivating the mutant allele while maintaining the wild-type *HTT*.	(186)
*HTT* non-allele (pan-HTT) disruption	*In vivo*: HD mouse models; *in vitro* neurons	CRISPR–Cas9 nuclease (disruption/indels)	AAV delivery to striatum/cortex (rodent); plasmid/RNP in cells	Complete disruption of *HTT* (both alleles) may diminish aggregates and enhance longevity and behavior in mice; nevertheless, it raises concerns with the loss of normal *HTT* function, which has been demonstrated to decrease inclusions and partially ameliorate abnormalities in certain studies.	(187)
CAG repeat excision/contraction (remove repeat tract)	Patient cells (fibroblasts, iPSC), *in vitro* neuronal derivatives	Paired-nicking/paired-Cas9 strategies to excise repeat region	Electroporation/ plasmid/RNP in cells (proof-of-concept)	Paired-guide methodologies may accurately remove the elongated CAG tract from exon 1 in patient cells, reinstating the normal reading frame or eliminating the deleterious repeat sequence, providing *in vitro* evidence that repeat removal is achievable.	(185)
CAG → CAA base editing (reduce uninterrupted CAG tract)	Patient cells and proof-of-concept mouse/cell models (recent studies)	Adenine and cytosine base editors (ABE/CBE) targeted within repeats to convert CAG to synonymous CAA codons	Plasmid/RNP/viral delivery in cells; experimental *in vivo* base-editing delivery in mice reported in preclinical studies	Transforming certain CAG codons to CAA diminishes the continuous CAG sequence (while preserving the encoded glutamine), which is anticipated and demonstrated to mitigate or postpone toxicity. Recent research illustrates molecular transformation and advantageous phenotypes in cellular structures and preliminary murine studies.	(389)
Prime editing for repeat correction (PE-CORE/repeat precise edits)	Cell models; method papers with mouse demonstrations for repeat diseases	Prime editing (PE) strategies adapted to correct or shorten repeat expansions (PE-CORE and related)	Plasmid/pegRNA delivery in cells; methods for *in vivo* pegRNA + PE machinery (AAV dual-vector) under development	Prime-editor methodologie, like PE-CORE, are being devised to accurately rectify repeat expansions (insertions/deletions or repeat contractions) without double-strand breaks; preliminary findings indicate programmable correction of repeats in cells, while methodological studies suggest *in vivo* translation.	(390)
Allele-specific PAM/SNP exploitation (PAM-altering SNPs, PAS)	Patient-derived cells, feasibility screens; *in vivo* allele-specific demonstrations in mice by SNP-guided strategies	CRISPR–Cas9 allele-specific editing exploiting PAM-altering SNPs or exon SNPs to selectively cut mutant allele	*In vitro*: electroporation/lipofection/ RNP; *in vivo* conceptually via AAV	The mapping of PAM-altering SNPs in patient populations facilitates the creation of allele-specific guides that exclusively cleave the *mHTT* allele. Monteys et al. and subsequent studies illustrate this as a viable approach for selective inactivation in several patients possessing the relevant SNPs.	(186)
iPSC isogenic lines & midbrain/cortical organoids (disease modeling and drug testing)	Patient iPSCs edited to create isogenic corrected/mutant lines; differentiated into neurons and organoids	CRISPR–Cas9 HDR for precise edits, CRISPRi for transcriptional modulation, base/prime editing for point conversions	Electroporation, RNP, or viral transduction at iPSC stage; subsequent differentiation to 2D neurons or 3D organoids	The isogenic correction of CAG length or SNPs in iPSCs rectifies cellular abnormalities, such as aggregation, transcriptional alterations, and neuronal degeneration. CRISPR-modified organoids replicate HD characteristics and serve as effective platforms for evaluating genome-editing strategies and small molecules.	(391)
Safety/long-term expression control (self-inactivating AAV, transient RNPs)	*In vivo* mouse models (recent preprints/methods)	Self-inactivating CRISPR constructs; transient RNP delivery; inducible systems	AAV designs that self-inactivate after editing; lipid nanoparticles and RNP electroporation for transient editing	Regulation of prolonged nuclease expression diminishes immunogenicity and the risk of off-target effects. Recent advancements in self-inactivating AAV designs and transitory RNP techniques	(392)
				enhance safety in preclinical models of Huntington's disease, as evidenced by recent preprints and methodological publications.	

HD, Huntington's disease; HTT, huntingtin gene; mHTT, mutant huntingtin; CRISPR, clustered regularly interspaced short palindromic repeats; Cas9, CRISPR-associated protein 9; iPSC, induced pluripotent stem cell; PAM, protospacer adjacent motif; SNP, single nucleotide polymorphism; PAS, PAM-altering SNP; KO, knockout; HDR, homology-directed repair; RNP, ribonucleoprotein complex; AAV, adeno-associated virus; PE, prime editing; pegRNA, prime-editing guide RNA; PE-CORE, prime-editing correction of repeat expansions; ABE, adenine base editor; CBE, cytosine base editor; CAG/CAA, trinucleotide codons encoding glutamine; 2D, two-dimensional cell culture; 3D, three-dimensional culture; ROS, reactive oxygen species.

Recent advancements indicate that CRISPR-Cas13 systems may be effective in treating HD by targeting RNA transcripts associated with the disorder. Morelli and Wu (152) conducted a notable study that advanced an enhanced CRISPR-Cas13d system known as Cas13d-CAGEX. This treatment led to enduring enhancements in motor function, reduced neuronal degeneration, and sustained therapeutic effects with minimal adverse effects observed for up to 8 months post-injection (200).

The efficient application of CRISPR-Cas13d demonstrates its considerable potential as an RNA-targeting tool for HD and possibly other dominant genetic disorders. Cas13d represents a safer alternative for prolonged gene regulation as it specifically inhibits pathogenic transcripts without altering genomic sequences. This differs from DNA-targeting techniques. Numerous studies employ CRISPR-Cas13 in Huntington's disease, although few utilize CRISPR-Cas12. No studies have utilized CRISPR-Cas3 in HD research, indicating that these methods remain underexplored for neurodegenerative therapeutics.

### 3.4 CRISPR-Cas in amyotrophic lateral sclerosis

ALS is a progressive ND characterized by the degeneration of both upper and lower motor neurons. This results in muscular weakening, atrophy, paralysis, and ultimately respiratory failure (201–203). Epidemiological studies indicate that incidence significantly varies by geography, with rates between 0.26 and 23.46 cases per 100,000 person-years, whereas prevalence ranges from 1.57 to 11.80 instances per 100,000 individuals across various countries (203, 204). Approximately 75% of ALS cases occur sporadically, although around 25% are familial and associated with over 135 genetic loci (205–208). A defining characteristic of ALS is the cytoplasmic mis localization and aggregation of TDP-43, which impairs RNA splicing, transport, and stability, resulting in extensive transcriptome dysregulation (202, 209). *Mutant superoxide dismutase 1 (SOD1) and fused in sarcoma (FUS)* intensify cellular stress through protein misfolding, oxidative injury, and impaired DNA damage repair. Intrinsic neuronal abnormalities are exacerbated by non-cell autonomous mechanisms, such as microglial activation, astrocytic glutamate toxicity due to diminished excitatory amino acid transporter 2 (EAAT2) expression, mitochondrial dysfunction, and compromised axonal transport. These mechanisms collectively begin a self-sustaining loop of neuroinflammation, energy depletion, and synaptic degeneration, ultimately resulting in motor neuron death (210, 211). The hexanucleotide (GGGGCC) expansion in C9orf72 is the predominant mutation among these. It induces toxic RNA foci and dipeptide repeat protein accumulation in around 40% of familial cases and 7% of sporadic cases (212–214). *TAR DNA-binding protein (TARDBP)* mutations disrupt TDP-43 protein homeostasis (4% familial, 1% sporadic) (215, 216), while *SOD1* mutations lead to oxidase dysregulation and protein aggregation (217, 218) in 12% of familial cases and 1%−2% of sporadic cases. Moreover, alterations in *FUS, heterogeneous nuclear ribonucleoprotein A1 (HNRNPA1), profilin 1 (PFN1)*, and *kinesin family member 5A (KIF5A)* influence RNA metabolism, axonal transport, and cytoskeletal architecture, ultimately resulting in motor neuron degeneration in ALS (219–223).

**Figure 3:**
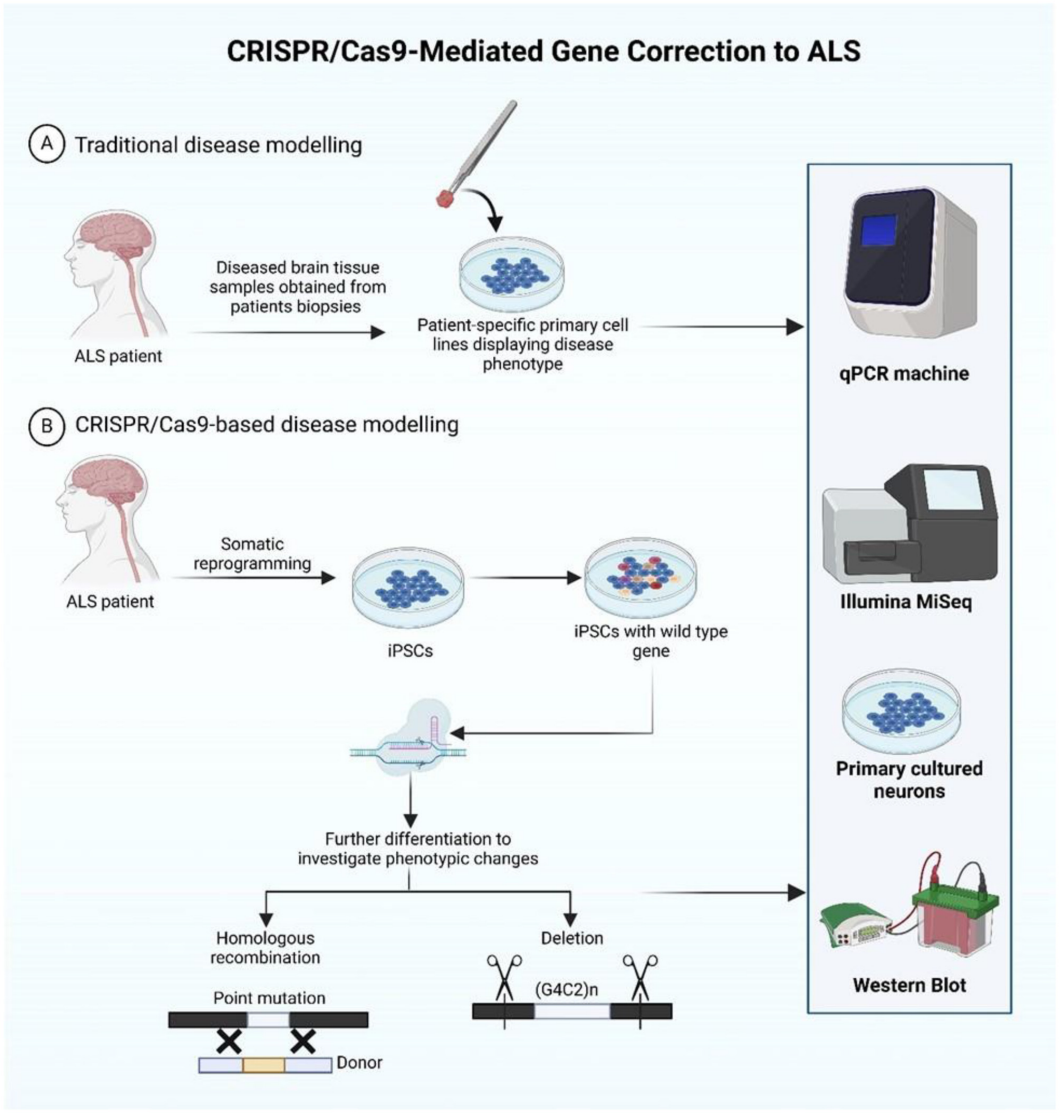
Schematic illustration shows the CRISPR-Cas9 uses in the investigation and treatment of Amyotrophic Lateral Sclerosis (ALS). **(A)** Traditional disease modeling involves obtaining diseased brain tissue biopsies to establish patient-specific primary cell lines. **(B)** CRISPR/Cas9-based disease modeling utilizes somatic reprogramming to create iPSCs, followed by gene editing (e.g., homologous recombination or deletion of G4C2 repeats) to investigate phenotypic changes in differentiated neurons. The figure was designed with BioRender (http://biorender.com/). CRISPR, clustered regularly interspaced short palindromic repeats; Cas9, CRISPR-associated protein 9; ALS, amyotrophic lateral sclerosis; iPSCs, induced pluripotent stem cells; qPCR, quantitative polymerase chain reaction ; G4C2, guanine—cytosine hexanucleotide repeat; MiSeq, Illumina sequencing platform.

The CRISPR–Cas genome-editing technology offers a significant method for developing targeted ALS therapies through precise genetic modifications (224). The protentional applications of CRSPR/Cas in ALS disease was gene silencing and correction, disease modeling, gene therapy delivery (16, 224–226) (Figure 3). *In vivo* CRISPR techniques have been employed to directly target pathogenic ALS mutations in animal models, yielding quantifiable functional improvements and molecular restoration. AAV-mediated CRISPR–Cas9 targeting of mutant *SOD1* decreased mutant SOD1 levels of proteins in the spinal cord and enhanced motor function and survival in transgenic mice, illustrating that somatic gene disruption can alleviate disease symptoms. Likewise, the excision of the pathogenic GGGGCC hexanucleotide repeat expansion (HRE) in C9orf72 by paired CRISPR–Cas9 guides diminished RNA foci and dipeptide repeat proteins, therefore ameliorating molecular abnormalities in various transgenic mice models (227). Recently, RNA-targeting CRISPR systems (Cas13/CasRx) designed for high fidelity have been utilized *in vivo* to diminish harmful repetitive RNAs and subsequent dipeptide repeat proteins (DPR) toxicity, providing a non-DNA-cleaving therapeutic approach that may mitigate irreversible on-target genetic concerns. These investigations demonstrate the feasibility of *in vivo* CRISPR interventions to reverse fundamental ALS processes, while also emphasizing the problems of delivery, immunogenicity, and product purity (editing byproducts) that persist for clinical application (227).

*In vitro* CRISPR modification of human cells, particularly patient-derived fibroblasts and iPSC-derived motor neurons, has proved essential for elucidating ALS pathophysiology and confirming targets. The CRISPR–Cas9-mediated removal or allelic modification of C9orf72 repetitions in patient-derived iPSC neurons diminishes RNA foci and DPR accumulation, while reinstating nucleocytoplasmic transport and cellular survival. CRISPR-induced knockouts of *SOD1, FUS*, or *TARDBP* in human cellular models replicate key mitochondrial, RNA metabolism, and proteostasis deficiencies, facilitating mechanistic comparisons among genotypes. The *in vitro* application of RNA-targeting Cas13 devices enables the direct reduction of hazardous repetitive RNAs in patient cells, facilitating swift assessments of guide efficacy and collateral activity prior to *in vivo* experimentation. Cell-based research is essential for determining dosage, assessing off-target effects, and developing biomarkers (228).

CRISPR–Cas9 technology has demonstrated its ability to rectify mutations associated with ALS in iPSC *in vitro* models derived from patient cells. For example, employing Cas9 along with a co-transfected donor template to rectify the *SOD1* A272C mutation in fibroblast-derived iPSCs restored the wild-type sequence (229). Similarly, rectifying the *SOD1* E100G mutation in iPSCs using homology-directed repair not only increased the quantity of motor neurons, the size of the soma, and the formation of neurites, but also diminished cell mortality post-differentiation (230). In C9orf72-associated ALS, employing CRISPR–CasRx to diminish sense and antisense repeat transcripts in HEK293T cells and patient-derived iPSC neurons decreased RNA foci and dipeptide repeat proteins, thereby providing protection against glutamate-induced excitotoxicity. Furthermore, utilizing Cas9 HDR to accurately excise the deleterious HRE from C9orf72 maintained the gene's integrity, restored expression and methylation levels to baseline, reduced intron retention, and eliminated detrimental repeat artifacts in motor neurons derived from iPSCs. All of these *in vitro* experiments demonstrate that CRISPR-based editing is a versatile and promising approach to address the primary genetic causes of ALS (231). The generation of SOD1+/A272C iPSCs and FUS+/G1566A iPSCs from primary fibroblasts of two ALS individuals, one possessing a heterozygous A272C mutation in the *SOD1* gene and the other a heterozygous G1566A mutation in the *FUS* gene. The CRISPR-Cas9 approach effectively rectified the SOD1+/A272C and FUS+/G1566A mutations in ALS iPSCs utilizing a donated plasmid or single-stranded oligodeoxynucleotide (ssODN) as the repair template, resulting in restored ALS iPSCs that exhibited normal pluripotency. Furthermore, SOD1+/A272C and repaired iPSCs were prompted to differentiate into motor neurons, resulting in the identification of 899 aberrant transcripts using RNA sequencing. This work may assist in identifying biomarkers and pathways for ALS and provides novel evidence for ALS therapy (229).

Base editing and CRISPR–Cas9 are two efficacious techniques for investigating and addressing ALS, particularly *in vivo* animals. An essential approach involves utilizing AAV9 to administer CRISPR components to transgenic ALS mice with the gain-of-function *SOD1* G93A mutation. Initial endeavors to employ *S. aureus* Cas9 (SaCas9) and gRNAs for enhancing survival and muscle preservation yielded limited success; hence, alternative strategies were explored. Base editors, including cytidine or adenine deaminase linked to a nickase Cas, enable accurate single-nucleotide modifications without generating double-strand breaks an advantageous characteristic for rectifying or deactivating point mutations in familial ALS. A pivotal proof-of-concept study using adenine base editing to inactivate a mutant *SOD1* allele *in vivo*, enhancing motor performance in an ALS mouse model. This demonstrated that base editing can effectively diminish hazardous protein levels and yield phenotypic advantages while circumventing several consequences associated with DSBs. Base editing is limited by editable sequence contexts and bystander edits, while the delivery of base-editor cargo to motor neurons presents a translational bottleneck; yet, these results demonstrate substantial potential for therapy for point-mutation ALS. Researchers employed a dual AAV9 split-intein methodology to circumvent the limitations of vector size, enabling the creation of specific nonsense mutations in *SOD1* (227, 232, 233). We must exercise caution in simultaneous initiatives aimed at targeting *FUS* and *TARDBP* mutations to avoid the detrimental consequences of entirely eliminating a gene (234, 235). Despite these challenges, employing CRISPR to reduce mutant *SOD1* in G93A-SOD1 mice significantly improved their motor functions, postponed illness onset by 37%, and extended their longevity by 25%. This indicates that gene editing possesses therapeutic potential (227).

CRISPR–Cas9 has been effectively employed to rectify faulty expansions in the C9orf72 gene, the most common genetic contributor to ALS. This pertains to SOD1-related ALS. Multiple groups have utilized paired guide RNAs around the hexanucleotide repeats to induce excision by NHEJ in iPSCs derived from patients. They have successfully eliminated up to 11% of the target DNA. Unanticipated deletions occurred; yet, C9orf72 expression remained constant (236–238). To enhance fidelity, normal-length repeats were included by HDR techniques, restoring typical methylation and transcription profiles. Neurons derived from these modified iPSCs exhibited a reduced number of RNA foci, diminished dipeptide repeat protein aggregates, and enhanced stress resilience (231, 239). SOD1 participates in the detoxification of superoxide radicals, and mutations in SOD1 lead to enhanced harmful activity (209, 240). The G93A-SOD1 mouse model possesses roughly 25 tandem repeat copies of the human SOD1G93A transgene, demonstrating signs of amyotrophy and dyskinesia observed in ALS patients (241). CRISPR modification of iPSCs has emerged as a fundamental element in ALS research. By producing isogenic iPSC pairs (mutant vs. corrected control), researchers can associate genotype with phenotype while minimizing background interference. The differentiation into motor neurons and three-dimensional spinal/brain organoids facilitates the replication of TDP-43 mislocalization, RNA foci, DPR synthesis, mitochondrial dysfunction, axonal transport abnormalities, and non-cell-autonomous contributions from astrocytes and microglia found in ALS. These CRISPR-iPSC platforms are extensively utilized for mechanistic analysis, high-throughput drug screening, and preclinical assessment of gene-editing methodologies, including the evaluation of AAV/SNP-guided allele selectivity, base editors, and Cas13 guides. iPSC platforms are essential for precise off-target and transcriptome-wide RNA-editing evaluations before animal or clinical use (226).

Recent advancements in CRISPR-based RNA-targeting methodologies suggest their potential utility in the treatment of ALS. Zhang et al. (2021) in their review article discussed an enhanced variant of CRISPR-Cas13d, termed CasRx, which can degrade both sense and antisense C9ORF72 repeat RNAs while preserving normal gene expression. Utilizing AAV vectors, CasRx was administered to HEK cells and patient-derived iPSC neurons *in vitro*, as well as to transgenic animal models *in vivo*. This resulted in significant reductions in RNA foci, DPRs, and glutamate-induced excitotoxicity (163). In another study, researchers employed Cas13 and Cas7-11 effectors to target ataxin-2 in models of TDP-43 proteinopathy. Direct injection of the Cas13 system into mice effectively reduced TDP-43 aggregation and the location of stress granules. This resulted in improved behavior, increased longevity, and reduced neurodegeneration in the mice. In comparison to previous Cas7-11 tools (242), Cas13 variants exhibiting enhanced fidelity demonstrated superior transcriptome specificity. The results indicate that RNA-targeting CRISPR systems possess significant potential as a therapeutic approach for ALS, as they can target critical disease characteristics. Table 5 illustrates CRISPR techniques in ALS.

**Table 5:** Applications of CRISPR–Cas in ALS.

**Gene target**	**Model**	**Editing/approach**	**Delivery vector/method**	**Result**	**Refs**.
*SOD1* (reduce toxic gain-of-function)	*In vivo* (*SOD*1^∧^G93A mice); *in vitro* patient cells and iPSC-derived motor neurons	CRISPR–Cas9 nuclease (inactivation) and adenine base editors (ABE)/cytosine base editors (CBE) for in-frame correction/premature stop introduction	AAV variants (AAV-PHP, AAV9) for brain/spinal delivery; split-intein base-editor AAV systems; RNP/plasmid for cells.	*In vivo* AAV-mediated delivery of CRISPR or base editors targeting human SOD1 diminished SOD1 levels, mitigated denervation and muscle atrophy, enhanced motor function, and prolonged survival in mouse models, demonstrating the therapeutic potential of genome editing of *SOD1* in mice. Base editors facilitated efficient inactivation while minimizing double-strand breaks (DSBs).	(367)
C9ORF72 (GGGGCC hexanucleotide repeat expansion)	*In vitro* patient fibroblasts/iPSC-neurons/ *in vivo* mouse models	CRISPR–Cas9 excision of repeat region (DNA) and CRISPR-Cas13/CasRx RNA targeting (to degrade repeat RNAs)	Cas9: plasmid/RNP in cells; AAV-Cas9 (or dual AAV) for *in vivo* excision in mice. Cas13/CasRx delivered via AAV or plasmid for RNA knockdown.	The Cas9-mediated excision of the repeat locus ameliorated critical disease mechanisms (decreased RNA foci, DPRs, and reinstated C9orf72 expression) in patient cells and *in vivo* models. The RNA-targeting Cas13/CasRx diminished harmful sense/antisense repeat RNAs and DPRs, safeguarding neurons in cellular and select *in vivo* experiments, thereby providing a nuclease-free, reversible alternative.	(228)
*TARDBP/TDP-43* (modeling & correction)	iPSC-derived motor neurons and isogenic iPSC lines; some organoid reports	CRISPR–Cas9 HDR for knock-in/knock-out and CRISPRi for modulation; creation of isogenic mutant and corrected lines	Electroporation/RNP/plasmid transfection at iPSC stage; lentiviral delivery for reporters; differentiate to MNs/organoids	CRISPR engineering of *TARDBP*, through the introduction or correction of disease mutations, produces iPSC-derived motor neurons that replicate TDP-43 mislocalization, aggregation, aberrant splicing, and neurodegenerative symptoms, serving as a robust platform for mechanistic studies and drug screening. Isogenic repair of *TARDBP* mutations ameliorates cellular abnormalities.	(393)
*FUS* (mutant *FUS*; stress granule dynamics)	iPSC-derived motor neurons, isogenic reporter lines; some *in vivo* modeling	CRISPR–Cas9 knock-in/knock-out to introduce *FUS* point mutations (e.g., P525L) and CRISPRi reporters	Electroporation/RNP/plasmid for iPSCs; lentiviral reporters; differentiate to neurons	Isogenic *FUS* mutant iPSC lines exhibit modified stress granule dynamics, FUS mislocalization, and increased neuronal susceptibility. CRISPR models facilitated the identification of modifiers and the evaluation of rescue techniques.	(394)
*ATXN2* (modifier of TDP-43 toxicity) — reduction as therapeutic strategy	CRISPR screens & iPSC models; preclinical ASO/AAV knockdown studies (modifier target)	Genome-wide CRISPR KO screens to find modifiers; CRISPR KO/knockdown of modifiers in cells	Pooled CRISPR libraries in cell lines/iPSC-neurons; AAV or RNAi/ASO used in preclinical modifier knockdown (*ATXN2*)	Genome-wide CRISPR screens found pathways and druggable targets (e.g., v-ATPase) and validated *ATXN2* as a modulator of TDP-43 toxicity. Decreasing *ATXN2* (via genetic methods or ASO/AAV RNAi) provides neuroprotection in animals, whereas CRISPR screens serve as an efficient tool to identify such targets.	(395)
RNA-targeting CRISPR (Cas13 family) for toxic RNAs/*DPRs*	*In vitro* patient iPSC-neurons and some *in vivo* mouse work in preclinical reports	CRISPR-Cas13 (CasRx/Cas13d) to degrade repeat transcripts and *DPR*-encoding RNAs	AAV or plasmid delivery; Cas13 systems are smaller (CasRx) and fit AAV packages better	Cas13d/CasRx addressing both sense and antisense strands C9orf72 Repeat RNAs diminish RNA foci, decrease DPR levels, and safeguard neurons from excitotoxicity in patient-derived neurons, while preliminary *in vivo* assessments suggest a viable RNA-centric alternative that circumvents DNA double-strand breaks. novel high-fidelity Cas13 variations reduce collateral activity.	(396)
Prime editing — precise correction (prospective/methods stage)	*In vitro* iPSC models; method papers discussing translation to CNS/ALS genes	Prime editing (PE) for precise single-base corrections (theoretical/applicable to point-mutation ALS genes)	Plasmid/pegRNA/PE protein in cells; dual-AAV dual-vector systems proposed for *in vivo* CNS	Prime editing offers the capacity to accurately rectify pathogenic *SOD1/FUS/TARDBP* point mutations without double-strand breaks; thus far, the majority of ALS-relevant prime edits have been proven in cellular models or addressed in methodological and review literature, indicating promise but still premature for substantial in-brain therapeutic results.	(397)
Organoids & complex human models (3-D motor neuron/spinal organoids)	iPSC → 3D motor neuron/spinal organoids edited by CRISPR to introduce/correct ALS mutations	CRISPR–Cas9 HDR/KO and CRISPRi performed at iPSC stage, then differentiated to organoids	Electroporation/RNP/ nucleofection at iPSC stage; subsequent 3D differentiation	CRISPR-edited organoids with C9orf72, *SOD1, FUS, or TARDBP* variations replicate RNA foci, DPRs, TDP-43 diseases, and neuronal degeneration with greater physiological relevance than two-dimensional cells, serving as models for mechanistic investigations and preclinical evaluations.	(228)
Safety/delivery strategies (transient RNPs, self-inactivating AAVs)	*In vivo* mouse models and method papers	Transient RNP electroporation; self-inactivating AAV designs; split-intein systems for large editors	LNPs (emerging), split-AAV, AAV-PHP variants, RNP electroporation in ex vivo cells	Transient and self-limiting distribution minimizes extended nuclease expression and immunological exposure; split/split-intein designs facilitate the delivery of base editors or prime editors to the CNS but remain in preclinical optimization. Delivery and immunological response constitute the primary translational obstacles.	(367)

ALS, amyotrophic lateral sclerosis; CRISPR, clustered regularly interspaced short palindromic repeats; Cas, CRISPR-associated (e.g., Cas9, Cas13); SOD1^∧^G93A, SOD1 glycine-to-alanine substitution at codon 93 (G93A) model; iPSC, induced pluripotent stem cell; MN(s), motor neuron(s); HDR, homology-directed repair; KO, knock-out; KI, knock-in; KD, knockdown; CRISPRi, CRISPR interference; ABE, adenine base editor; CBE, cytosine base editor; DSB(s), double-strand break(s); AAV, adeno-associated virus (e.g., AAV9; AAV-PHP variants); RNP, ribonucleoprotein (Cas protein + guide RNA complex); ASO, antisense oligonucleotide; RNAi, RNA interference; DPR(s), dipeptide repeat protein(s); CasRx/Cas13d, Cas13d (also called CasRx) RNA-targeting CRISPR effector; PE, prime editing; pegRNA, prime-editing guide RNA; PBS, primer-binding site; PAM, protospacer adjacent motif; v-ATPase, vacuolar-type H+-ATPase; CNS, central nervous system; LNP(s), lipid nanoparticle(s).

## 4 Challenges and strategies for applying CRISPR–Cas in treating neurodegenerative disorders

### 4.1 Delivery mechanisms

The efficient and selective delivery to certain cell types is the primary practical limitation for CNS-targeted CRISPR therapies. AAV vectors provide strong neuronal tropism, sustained transgene expression, and established manufacturing processes, rendering them the primary tool for *in vivo* CNS editing; however, their restricted packaging capacity hinders the delivery of larger editors (such as prime editors and certain base-editor constructs), and continuous Cas expression from AAV may elevate off-target activity and immunogenicity (243). Lipid nanoparticles (LNPs) offer a supplementary, transient-expression approach for delivering mRNA or RNPs, thereby minimizing the editor's exposure duration. Recent advancements in LNP formulations have facilitated systemic and targeted delivery in preclinical models; however, efficient BBB penetration and neuronal specificity continue to be areas requiring further optimization (244). Engineered extracellular vesicles/exosomes represent a novel option characterized by low immunogenicity and potential for targeted cell delivery; ongoing research emphasizes scalable loading techniques, surface modification for tropism, and comprehensive biodistribution characterization (245). A pragmatic hybrid approach such as compact editors encapsulated in AAV for localized distribution, LNPs for temporary systemic administration, or exosome-based vehicles.

#### 4.1.1 Challenge

**Figure 4:**
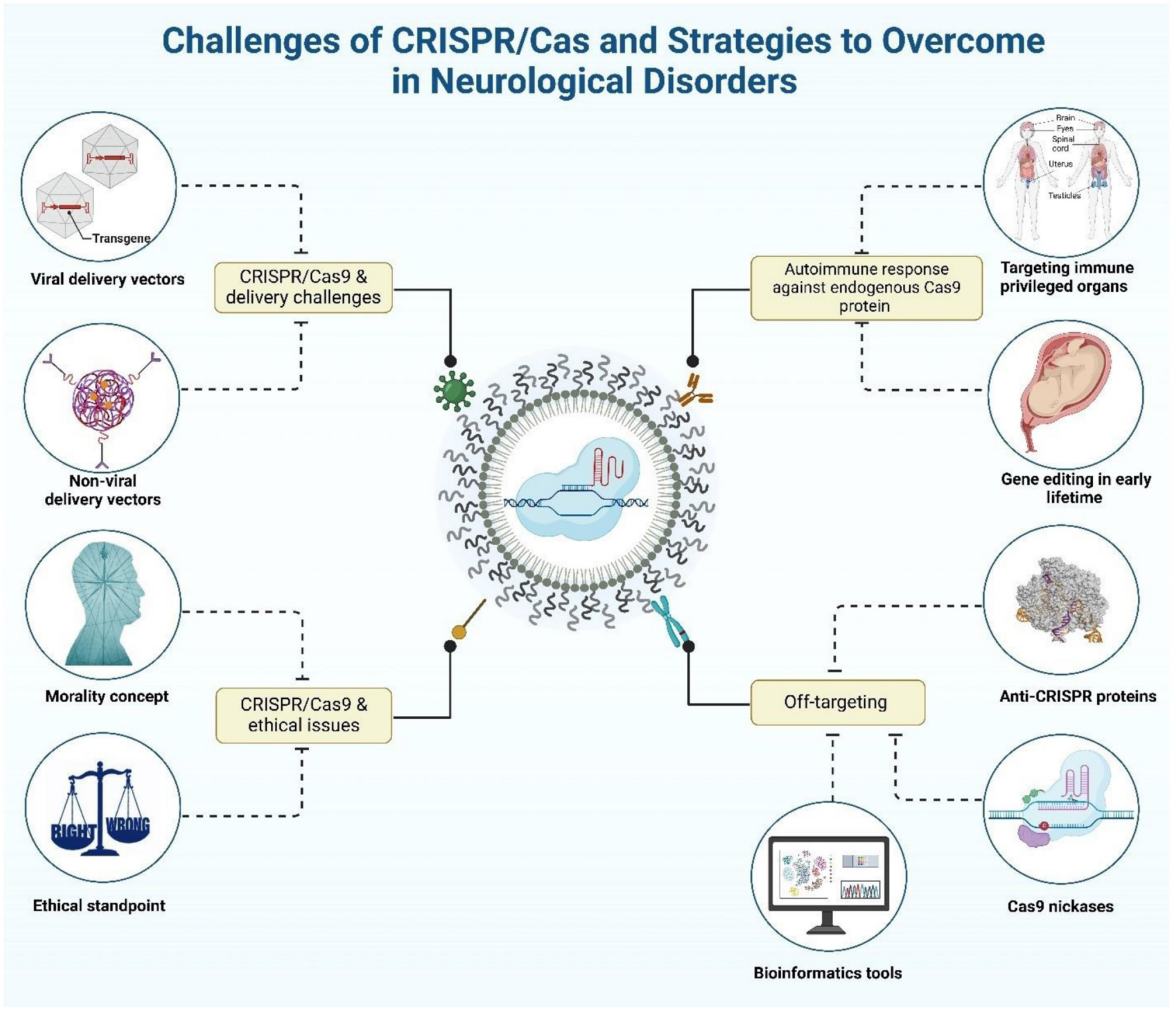
The schematic diagram shows the analysis of the challenges in implementing CRISPR–Cas9 technology for neurological illnesses and approaches to mitigate these challenges. The figure was designed with BioRender (http://Biorender.com/). CRISPR, clustered regularly interspaced short palindromic repeats; Cas9, CRISPR-associated protein 9.

Investigating the delivery of the CRISPR–Cas system for the treatment of NDs necessitates the resolution of significant technical challenges to ensure safety and efficacy (Figure 4). The CNS is safeguarded by the BBB, which serves as a selective permeability barrier to limit the entry of chemicals from the bloodstream into brain tissue (246, 247). The protective function of the BBB presents significant challenges for administering CRISPR–Cas9-based therapies for neurodegenerative diseases. The dimensions of the CRISPR–Cas9 complex hinder its ability to traverse the BBB due to its substantial size. Passive diffusion seems to be the only process by which small lipophilic molecules traverse the barrier, but active transport is essential for the movement of bigger entities. The delivery of CRISPR–Cas9 tools encounters obstacles resulting from their dimensional size (248, 249). The BBB inhibits the ingress of external substances into the brain, encompassing pathogens, and toxins (250). The CRISPR–Cas9 complexes are unable to traverse the BBB due to its specialized mechanisms for critical nutrients and chemicals, which do not accommodate the transit of big macromolecules. Owing to the restrictive properties of the BBB, researchers must develop specialized delivery systems that facilitate the successful passage of gene-editing tools past this barrier (251).

Researchers employ AAVs as vectors for delivering CRISPR components due to their outstanding efficacy and low safety risks. AAVs possess limited capacity for packing materials, complicating the simultaneous delivery of Cas9 proteins and their guide RNAs. The utilization of AAV vectors for the delivery of CRISPR components encounters numerous constraints, as capsids elicit immunological responses, leading to off-target mutations, and AAV distribution may be detrimental to cellular DNA (252, 253). Although viral vectors are successful, they elicit immunological responses that may provoke undesirable consequences in the body. Research has demonstrated that viral vectors induce unwanted mutations and severe immune responses, rendering them unacceptably hazardous for live human use (254). Non-viral delivery approaches utilizing nanoparticles offer safer options, although they encounter distinct challenges. The effectiveness of BBB transport and neuron-specific targeting continue to be significant obstacles hindering successful research. The reliable transport and regulated release of CRISPR components require enhanced optimization for success (255, 256).

#### 4.1.2 Potential strategy

The strategies overcome challenges for BBB, engineered variants of AAVs have demonstrated enhanced capabilities for penetrating the BBB, as researchers have tailored them for gene therapy applications aimed at treating neurodegenerative diseases (Figure 4). Researchers have modified AAV capsid proteins to enhance their affinity for endothelium receptors, hence facilitating receptor-mediated transcytosis. Experimental results demonstrated that the Cre recombinase-based AAV targeted evolution (CREATE)-derived AAV-PHP.B variant surpassed AAV9 in CNS transduction efficacy by a factor of 40 following intravenous administration in adult mice. Researchers enhanced AAV-PHP.eB to achieve improved CNS transduction and BBB penetration efficacy following its first creation. Administration of AAV-PHP.eB via the intravenous method in adult mice resulted in neuronal transduction of 69% of cortical cells and 55% of striatal cells with a vector genome dosage of 1 × 10^11^. Engineered AAVs can function as effective non-invasive delivery methods for genetic therapies targeting NDs (257, 258).

Researchers are developing nanoparticles to stabilize CRISPR–Cas9 components and ensure their safe transit across the BBB. The administration of mRNA-based therapeutics to the brain is effectively achieved via LNPs that facilitate the passage of genetic material across BBB (249, 251, 259, 260). The functionalization of LNPs with transferrin enhances their ability to traverse the BBB. Brain endothelial cells exhibit elevated numbers of transferrin receptors, which utilize their inherent iron transport capability for receptor-mediated transcytosis of transferrin-conjugated nanoparticles. This technique enables the delivery of medicinal medicines to the brain. A document delineates nanoparticle targeting of the brain by transferrin nanoparticle conjugation (261). Exosomes, as natural extracellular vesicles, exhibit enhanced brain-targeting capabilities when suitably modified. The incorporation of transferrin or specific peptides onto exosomes facilitates their binding to receptors located on the BBB for targeted CNS delivery. Modified exosomes displaying transferrin on their surface enhance the accumulation of these vesicles in brain tissues, facilitating more effective administration of therapeutic agents. Ongoing research indicates that exosomes exhibit enhanced efficacy for CNS distribution when modified with ApoB to interact with low-density lipoprotein receptors (LDLR) (262).

Therapeutic medicines are delivered via intracerebral injection straight into specific brain regions for optimal distribution. This delivery technique improves the localized dispersion of CRISPR–Cas9 components, potentially increasing their concentration at the target region. The administration of therapeutic agents by intracerebral delivery enables healthcare professionals to accurately inject these substances into targeted brain regions, ensuring optimal distribution. This technique facilitates local delivery of CRISPR–Cas9 components, enhancing distribution and potentially increasing their concentration at the target region (263). Another study employed biodegradable PEGylated nanocapsules to provide Cas9-sgRNA ribonucleoproteins (RNPs) during intracerebral injections into the mouse striatum. This therapeutic approach achieved precise genome alteration in medium spiny neurons with minimal stimulation of glial cells, ensuring safety (264). The direct delivery methods present intriguing strategies for CRISPR–Cas9-mediated therapeutic interventions in NDs. Further issues regarding surgical invasiveness, tissue damage mitigation, and precise targeting criteria must be addressed to improve the clinical application of these techniques.

Another strategy was utilized PepFect14, in conjunction with a portion of the rabies virus glycoprotein, was utilized to create cell-penetrating peptides that associate with CRISPR–Cas9 components for the targeting of neuronal cells. The approach emphasizes receptor-mediated transcytosis to facilitate the passage of gene-editing tools across the BBB for transport (260, 265–267).

### 4.2 Off-target effects

Long-term safety issues associated with CRISPR encompass not just off-target mutations but also significant on-target deletions, insertions, inversions, chromosomal translocations, and other intricate structural changes that conventional PCR techniques may overlook. Identifying these occurrences necessitates genome-wide, high-resolution methodologies, including long-read sequencing and capture-based assays. Strategies for risk mitigation encompass the temporary administration of CRISPR components (RNP or mRNA), the utilization of high-fidelity or double-strand break-free editors (nickase, base, or prime editors), and meticulous optimization of guide RNA, bolstered by empirical off-target analysis. Comprehensive preclinical genotoxicity assessment and prolonged molecular and clinical surveillance, with established cessation criteria, are crucial for initial human studies (268).

#### 4.2.1 Challenge

The occurrence of off-target cleavage has the potential to lead to unfavorable mutations and cytotoxicity, which may disturb the regular functioning of genes (269). The off-target effects linked with sgRNA and Cas9 pose significant concerns regarding the CRISPR–Cas method (Figure 4). While Cas9 can tolerate variations in the distribution and quantity of mismatches in DNA, the brevity of sgRNA (about 17–24 bp) heightens the likelihood of off-target effects. The presence of similar sequences or sequences with minor variations elsewhere in the genome can lead to mistargeting due to slight variances in the sgRNA (270–272). Mis-targeting may result in severe implications for patients, including the development of oncogenic mutations (273). CRISPR–Cas9 randomly alters tumor suppressor genes and activates oncogenes, potentially leading to cancer development. Off-target effects occur with a significant frequency of 50% every study, raising considerable concerns for therapeutic application (274). The incidence neuronal dysfunction of inadvertent genetic alterations due to off-target impacts jeopardizes critical neurological genes, potentially leading to neuronal cell disorders. Off-target action occurs regularly, highlighting the necessity for scientists to implement precise targeting techniques to mitigate these adverse outcomes (275, 276).

Creating sgRNA with high specificity and an allowable base mismatch to another sequence reduces the likelihood of mis-targeting sgRNA. The insufficient understanding of CNS biomarkers is a significant obstacle to effective cell targeting and signaling responses, yet advancements in CSF biomarkers, brain imaging, and varied vector modeling are expected to enhance specificity (277).

#### 4.2.2 Potential strategy

An optimal sgRNA length, minimal mismatches, and optimum GC content can enhance the targeting efficiency of CRISPR-Cas9 technology. Ren et al. (269) demonstrated that sgRNA sequences ranging from 17 to 20 bp have comparable mutation efficiencies. Fu et al. (278) discovered that shortened gRNA sequences of 17 or 18 bp enhance specificity, while sgRNA sequences with a GC concentration of 40%−60% are advantageous for augmenting target effects (269, 279). Another strategy to reduce off-target effects is to select a low GC content of sgRNA; decreased GC content correlates with diminished stability of nucleic acid hybrids. Besides screening for distinct genomic sequence targets, modifying Cas9 enzymes to create nickases and fusing dCas9 with the FokI catalytic domain has diminished the incidence of off-target effects in CRISPR–Cas9 applications (273). The sgRNA specificity, particularly with fewer than three mismatches within 10–12 base pairs close to the PAM site and more than two mismatches with the off-target site, can enhance targeting efficacy (275, 280). Additionally, preventing the emergence of sgRNA bulges at 12 bp next to the PAM site, generating chimeric sgRNA, substituting portions of the RNA sequence with a DNA sequence, and employing numerous sgRNAs directed at the same gene may also diminish off-target effects (280–283). Various variants of CRISPR–Cas9 could affect and raise the target accuracy of CRISPR technology, engineered a high-fidelity version 1 of SpCas9 (SpCas9-HF1) by Kleinstiver et al. (284). In comparison to wildtype SpCas9, SpCas9-HF1 has similar targeting efficacy. Moreover, SpCas9-HF1 possesses the ability to diminish non-specific DNA interactions, rendering genome-wide break capture and focused sequencing techniques incapable of identifying all or nearly all target activities (284). Developed an “enhanced specificity” SpCas9 variant 1.1 (eSpCas9 1.1). In comparison to wildtype SpCas9, eSpCas9 1.1 demonstrates robust on-target cleavage and significantly reduced effects beyond the target (285). Developed an innovative hyper-accurate CRISP/Cas9 variant (HypaCas9) that demonstrated elevated genome-wide specificity without compromising on-target activity. Moreover, SpCas9 nickase, Sniper-Cas9, and xCas9 variants can reduce off-target effects in genome editing to differing extents (276, 286).

Both base editing and prime editing variants of CRISPR based genome editing technologies facilitate precise DNA changes by eliminating the formation of DSBs, hence mitigating the risk associated with unintended insertions or deletions (indels). Effective methodologies are now available to mitigate the unexpected consequences that conventional CRISPR–Cas9 editing may induce in DNA sequences (287). Base editors provide genomic location-specific DNA base changes directly and irreversibly, while avoiding the creation of double-strand breaks. The approach mitigates the danger of generating indels due to its architecture. The research indicates that base editors induce unintended alterations in DNA and RNA due to their off-target activity. Scientific research indicates that deaminase enzymes employed in CBEs and ABEs result in significant unintentional genomic modifications due to their interaction with various DNA bases (287, 288). Investigative research indicates that prime editing effectuates precise modifications via an alteration of reverse transcriptase and a compromised Cas9 endonuclease, directed by pegRNA (289). A research study demonstrated the efficacy of prime editing in introducing the AD-associated *APOE4* mutation into human induced iPSCs with high precision and productivity, resulting in editing success rates of 28.2% in iPSCs and 42.3% in HEK293T cells. The approach exhibited no indication of significant unwanted direct alterations at the target location and no remarkable off-target modification effects, so rendering it appropriate for development (99).

The CRISPR–Cas9 genome editing technique encounters significant challenges due to off-target effects, which can result in unintended alterations to the genetic material. Bioinformatics tools have been created to enhance sgRNA design and assess off-target activity. DeepCRISPR and GUIDE-seq serve as crucial tools that augment CRISPR-based applications by enhancing their specificity and safety (50, 290).

DeepCRISPR employs deep learning technology to create an optimization framework that forecasts target-binding efficacy and undesirable DNA interactions of sgRNA. The integration of nucleotide sequences and epigenetic characteristics across several human cell types via DeepCRISPR yields a comprehensive assessment of sgRNA efficacy. This method enables scientists to identify sgRNAs that demonstrate robust on-target efficacy while minimizing off-target effects, thereby enhancing the precision of CRISPR–Cas9 editing (291). GUIDE-seq is an experimental method that enables genome-wide identification of double-stranded breaks caused by CRISPR-Cas nucleases through a sequence-based technique. The sequencing of double-stranded oligodeoxynucleotides incorporated into double-strand breaks enables GUIDE-seq to produce comprehensive off-target site data. The procurement of this data allows researchers to observe unintentional genetic alterations that transpire throughout CRISPR-based initiatives (50, 292).

### 4.3 Immune response

Both the adaptive and innate immune responses present significant challenges for repeated or systemic CRISPR therapy. Documented pre-existing humoral and cellular immunity to AAV capsids and frequently utilized Cas orthologs (SpCas9, SaCas9) can neutralize vectors or induce T-cell-mediated clearance of edited cells; additionally, sustained expression of bacterial nucleases extends the period for immune recognition (293). Effective mitigation strategies, substantiated by preclinical and early clinical studies, encompass the utilization of less common AAV serotypes or engineered capsids, transient immunosuppression during dosing, the administration of editors as mRNA or RNPs (via LNPs or alternative transient carriers) to minimize antigen exposure, and the creation of Cas variants featuring diminished immunogenic epitopes or humanized sequences. Thorough immune profiling in pertinent animal models and patient samples is essential for formulating appropriate therapeutic regimens (294).

Importantly, although pre-existing humoral and cellular immunity to commonly used Cas orthologs (e.g., SpCas9/SaCas9) has been documented in human populations (295), published clinical experience with CRISPR–Cas9 therapies to date has not reported clinically meaningful Cas9-directed immune toxicities (296). For example, in the first systemic *in vivo* CRISPR–Cas9 trials targeting transthyretin (TTR) (LPN delivery of Cas9 mRNA and sgRNA; NCT04601051), reported treatment-related events were primarily transient infusion-related reactions (and transient liver-enzyme elevations in some participants), without persistent immune-mediated sequelae attributed specifically to Cas9 (297). In parallel, *ex vivo* CRISPR–Cas9 edited autologous hematopoietic stem and progenitor cells products (e.g., Exagamglogene autotemcel) have shown safety profiles largely consistent with conditioning/transplant procedures rather than editor immunogenicity, which is biologically plausible given the transient exposure to Cas9 during cell manufacturing (298).

Nevertheless, ongoing and future trials, particularly those requiring CNS delivery and/or potential redosing, should continue systematic immune surveillance (anti-Cas antibodies, T-cell responses, cytokine profiles) to define risk more precisely in neurological indications.

#### 4.3.1 Challenge

Individuals possessing preexisting immunity to Cas9 derived from bacterial sources such as *Staphylococcus aureus* and *Streptococcus pyogenes* diminish the efficacy of CRISPR–Cas9 gene editing therapy (273, 299). The research analysis identified SaCas9 antibodies in 78% of participants and SpCas9 (*S. pyogenes* Cas9) antibodies in 58%. Laboratory analyses revealed that SaCas9 antibodies were present in 78% of examined donors, whilst SpCas9 antibodies were found in 67% of individuals. Research findings indicated a significant number of T cells in the adult human population that interacted with SpCas9 (300, 301). Patients' previous immune responses often swiftly eliminate cells via Cas9 expression, diminishing therapeutic efficacy and potentially inciting inflammatory reactions or negative outcomes. The resolution of this issue is crucial for physicians to appropriately utilize CRISPR–Cas9 technology in medical applications (27, 300, 302).

Furthermore, the delivery mechanisms of CRISPR systems can stimulate immunological responses and affect neural cells. Viral vectors are frequently employed to transport CRISPR constructs across the BBB (303, 304). Nonetheless, viral transmission results in prolonged CRISPR expression, which is detrimental to neuronal cells and modifies in phenotypes of neuronal (303). Furthermore, chronic production of Cas9 induces a cytotoxic immune system response that eliminates genetically modified cells. The elimination of changed neurons in the brain may result in detrimental effects, as neuronal regeneration is restricted (294). The implementation of CRISPR–Cas9 technology is hindered by the immunological response triggered by the Cas9 protein or delivering vectors in the host (300). The gene therapy vectors utilizing AAV frequently employed in clinical applications elicit a cytotoxic T-cell response, impacting both their safety and efficacy results (Figure 4). AAV capsid proteins introduced into the body are subjected to natural degradation processes, enabling major histocompatibility complex MHC class I molecules to present these components on the surfaces of transduced cells. The activation of cytotoxic T lymphocytes (CTLs) transpires via this presentation pathway, leading to cellular death and a subsequent decrease in transgene expression (305–307). Three categories of T-cell responses in patients are contingent upon critical factors from AAV capsids, vector dose, and the patients' immunological backgrounds, including their natural exposure to wild-type AAV (305). Comprehending and mitigating these immunological barriers is essential for improving the safety and efficacy of AAV-mediated gene treatments (306).

#### 4.3.2 Potential strategy

Various strategies have been devised to diminish the immunogenicity of CRISPR-Cas9 systems for therapeutic applications. Acquiring CRISPR from non-pathogenic bacterial strains offers a strategy to mitigate potential risks associated with pre-existing immune responses. Studies indicate that Cas9 derived from *Geobacillus stearothermophilus* (GsCas9) exhibits superior stability in human plasma compared to *Streptococcus pyogenes* Cas9 (SpCas9), suggesting its potential as a less immunogenic method for genome editing (299, 308). Another strategy is to target modifications of Cas9 immunogenic epitopes by engineering techniques yield less detectable proteins that preserve their editing capabilities. Researchers identified significant T-cell antigenic sites in SaCas9, after which other modifications were introduced to reduce immunogenicity (302, 309). Proposed alterations in these engineered versions resulted in a diminished T-cell response while maintaining their inherent nuclease activity (310, 311). The delivery of CRISPR-Cas9 components via mRNA results in a temporary impact that mitigates immune responses, hence enhancing the safety of genome editing therapies (312). The utilization of Cas9 mRNA facilitates temporal protein expression, hence minimizing prolonged exposure and decreasing vulnerability to immune responses. Target cells efficiently acquire Cas9 mRNA and sgRNA via LNPs, which maintain transient protein expression and mitigate undesirable immunological responses (313). This approach yields superior outcomes compared to traditional viral vectors, as it minimizes genetic changes and allows for numerous distribution opportunities due to its diminished inflammatory response. Research indicates that employing LNP to deliver Cas9 mRNA facilitates genome editing throughout many administrations with less immune response upon delivery (314). Modified nucleoside addition to mRNA molecules is extensively employed by scientific researchers as an established technique for developing safer CRISPR-based therapy. Researchers found that including pseudouridine and 5-methylcytosine into Cas9 mRNA significantly diminished the immunological response, hence enhancing Cas9 protein synthesis (315). Research has demonstrated that substituting uridine with N1-methylpseudouridine in mRNA vaccines diminishes immune responses while enhancing protein synthesis (316). The therapeutic efficacy improves as these changes allow mRNA to evade immune recognition, hence reducing unfavorable immunological responses (317).

Administering short-term immunosuppressive regimens with CRISPR–Cas9 delivery may reduce immune responses; nonetheless, this strategy is contentious and requires comprehensive safety assessments (318). Research has shown that the immune system can identify and respond to CRISPR elements, resulting in inflammation and diminished effectiveness of gene editing (302). Research demonstrates that Cas9 expression in murine muscle tissue led to lymphocyte infiltration, implying an immunological response to the exogenous protein (319).

### 4.4 Ethical and regulatory challenges

CRISPR-based therapeutics for NDs presents significant ethical and regulatory issues, especially when utilized solely on somatic cells. Critical concerns encompass securing informed consent for permanent genomic modifications, equitable access to expensive therapies, and meticulously assessing the risks and benefits of irreversible operations in the CNS. First-in-human trials necessitate stringent supervision, particularly when employing novel delivery technologies. Despite widespread rejection of germline editing, its public discourse continues to influence trust in somatic editing. Robust community involvement, autonomous ethical assessment, and compliance with international standards are needed. Regulators are progressively mandating comprehensive preclinical evidence about safety, immunological responses, and off-target effects, in addition to dependable manufacturing and long-term monitoring strategies. Engaging bioethicists and patient representatives in trial design enhances the quality of consent and public acceptance (320).

#### 4.4.1 Challenge

CRISPR gene-editing in neurological research raises significant ethical issues owing to its substantial influence on cognitive abilities and identity, possible unforeseen consequences, and ramifications for subsequent generations.

Ethical considerations are crucial, considering the fragility of several patients, and acquiring informed approval. Consent may be more complex due to cognitive problems. Regulatory obstacles in guaranteeing the safety of experimental therapies, while accelerating approvals for life-threatening disorders, contribute to the complexity (321).

The application of gene-editing technologies in the brain poses risks of off-target effects that may disrupt normal neural processes, potentially resulting in alterations in behavior or cognitive capacities. The intricacy of the human brain renders such risks perilous, since little alterations from a brain computer interface could lead to substantial neurological repercussions that may not become apparent until later in life. These warrants worry if the targeted modification involves germline cells, as they transmit genetic mutations to subsequent generations, hence amplifying the potential repercussions of such changes indefinitely (322). The intentional alteration of DNA in human embryos, gametes, and zygotes for the aim of disease prevention has sparked significant ethical controversies (323, 324). The major ethical dilemma arises from the capacity to engineer “designer babies” by DNA alteration, which pursues medicinal advantages while also striving to cultivate enhanced inherited traits, so provoking concerns over eugenics and inequality (325). Opponents argue that these methods would create communities with genetically modified elites, exacerbating existing societal disparities (323). Ethical considerations are crucial prerequisites prior to the application of CRISPR in humans. Genome editing in clinical contexts is still restricted to somatic cells, as this poses a lower risk of unethical exploitation compared to germline modification (326). Germline editing raises ethical dilemmas because of its incalculable implications over generations (323). Modifications made during the embryonic period are heritable, indicating they will be passed on to subsequent generations. Such erratic alterations pose risks that impact particular people and future generations within the human gene pool (327). The uncertainties associated with mutations and unanticipated adverse effects from these therapies raise questions regarding their safety and efficacy (328). Germline editing encounters substantial difficulties due to the complexities of obtaining consent. Individuals who have not yet been born cannot consent to genetic alterations performed prior to their birth. Scientists encounter an ethical quandary over their authority to implement irreversible alterations on individual's incapable of providing consent for such adjustments. Individuals subjected to germline editing experience a restriction of their autonomy rights, so limiting their capacity to make decisions regarding significant life-altering issues (329, 330).

However, the practice of germline gene editing holds potential for preventing specific genetic abnormalities of the nervous system, although it engenders significant ethical issues. Ethical frameworks and comprehensive evaluations must be established to address concerns around designer babies, unforeseen consequences, issues of consent, and potential misuse prior to the ethical application of such technology.

In addition, the ethical considerations for genome editing fundamentally differ between somatic and germline operations. Somatic editing, which focuses on non-reproductive cells, aligns with conventional therapeutic ethics when risks, benefits, and informed consent are adequately addressed (331). In contrast, germline or heritable editing presents intergenerational, societal, and justice issues, as modifications would be passed to future generations and impact individuals unable to provide consent (332). International expert organizations and policy evaluations consistently advise against permitting clinical germline heritable genome editing outside of stringent research frameworks at this time, asserting that any future deliberation necessitates exceptional evidence of safety, efficacy, and widespread societal agreement. The updated wording encapsulates these viewpoints and emphasizes the practical ramifications for researchers and sponsors: prioritize robust somatic-cell methodologies for imminent clinical application while fostering responsible, transparent research about long-term ethical considerations (332).

A verified translational pathway commences with mechanism validation in pertinent models (isogenic human cell structures, organoids, and various animal species), coupled with stringent safety assays (empirical genome-wide off-target mapping, long-read structural variant detection, transcriptomic profiling, and immunogenicity testing) (333). Investigational New Drug/Clinical Trial Application IND/CTA enabling packages must encompass biodistribution, vector shedding, reproductive toxicity, and dose-ranging investigations. Manufacturing must adhere to good manufacturing practice (GMP) standards and incorporate confirmed potency assays associated with the editing method; for viral vectors, capsid characterization and release assays are crucial. Initial clinical trials must utilize careful, incremental dose escalation, stringent molecular monitoring of editing results in accessible tissues, clinical indicators for target engagement, and established stopping criteria. Sponsors must ultimately engage in long-term follow-up (multi-year, disease-specific) to identify delayed side effects and gather enduring efficacy data. These measures correspond with the recommendations of international commissions and recent translational assessments (334).

The brain's CRISPR technologies raise pressing issues related to rational agency and voluntariness. The dilemma of who should provide permission for a person's personality to change arises if cognitive qualities can be altered by gene editing. This is particularly difficult when a subject is unable to make educated decisions for various reasons, such as mental impairments (335, 336).

Ethical considerations around brain somatic gene editing arise due to the potential for unintended genetic modifications that may be inherited by subsequent generations. The targeted modifications of non-reproductive cells may indirectly induce changes in germline cells, potentially resulting in irreversible genetic abnormalities. Somatic gene editing raises ethical dilemmas about permission, as future generations are unable to provide assent for such alterations (331, 337). Although somatic edits are generally non-heritable, there is apprehension that unintended alterations might be transmitted to future generations if specific editing inadvertently impacts germline cells or influences mechanisms affecting genetic material in incompletely understood ways. This issue highlights ethical dilemmas concerning the limits of human control over gene editing, particularly in relation to somatic therapies designed to treat NDs (338–340). The difficulty in obtaining consent for brain somatic gene editing remains significant within children and cognitively challenged patient demographics. An ethical dilemma arises since stakeholders involved in gene editing interventions may be unaware of the various risks and genetic alterations that could come from these procedures (341, 342). Somatic gene editing in the brain may result in unintended consequences owing to the intricacy of neural circuitry and cerebral function. The ethical dilemma pertains to the potential risks of modifying the brain's genetic composition without a comprehensive understanding of the long-term safety of such operations. Even slight, inadvertent alterations in genetic material may result in significant NDs or unexpected psychological diseases (343, 344).

#### 4.4.2 Potential strategy

Human gene editing utilizing CRISPR has raised significant concerns, particularly regarding germline modifications (345). Current guidelines, including those from the national institutes of health (NIH) and World Health Organization (WHO), emphasize restricting CRISPR treatments to somatic cells in adults to prevent heritable alterations that may inadvertently affect subsequent generations (Figure 4) (346, 347). By focusing on non-reproductive cells, somatic gene editing mitigates any ethical dilemmas associated with unintended genetic alterations (331). The formulation of these regulations primarily relies on comprehensive risk evaluations and continuous monitoring to avert the misuse of gene-editing technology (348). The NIH has instituted stringent standards for clinical research utilizing gene-edited technology, necessitating comprehensive assessment by institutional ethics committees (344, 349). Various nations implement distinct policies; in China, research on CRISPR technology progresses with fairly lenient regulations, whereas Western countries enforce stringent restrictions governing human gene editing (350, 351).

Reaching an international agreement on CRISPR guidelines is vitally vital given the global nature of scientific collaboration (352). The World Medical Association (WMA) has recommendations meant to guide members in creating consistent standards for CRISPR research (353). These requirements underscore the necessity of global control in order to stop unethical behavior and guarantee responsible and safe evolution of gene-edited technology. Harmonizing guidelines will assist in stopping “ethics shopping,” in which researchers may undertake experiments in countries with more lax laws, therefore avoiding more rigorous ethical rules elsewhere (354, 355). Moreover, a combined worldwide framework would help to improve cooperation, enable more effective monitoring, and advance equity in access to contemporary medical treatments (349). Robust ethical norms and international cooperation mitigate the risks associated with CRISPR technology and ensure its appropriate use for societal benefit.

## 5 CRISPR-based therapeutics for neurological disorders in clinical trials

The neurological disease clinical trials provide distinct obstacles owing to the complexity and variability of these conditions. A major problem is patient recruitment, as identifying appropriate individuals with certain NDs might be arduous. Moreover, the advancement of NDs differs significantly among people, complicating the establishment of standardized trial endpoints (321). No clinical trials utilizing CRISPR for NDs have commenced yet. The majority of current clinical trials focus on cancer treatment and immunotherapy. CRISPR-mediated modification of T-cell antigen receptors (CAR-T technology) is being investigated for the treatment of multiple myeloma, sarcoma, and melanoma in the United States (356). A new clinical CAR-T trial has commenced in China for lymphoma, B-cell leukemia, invasive bladder cancer, and metastatic non-small-cell lung carcinoma (NSCLC) (357).

Some clinical trials have been recorded in http://clinicaltrials.gov applied on neurological disease such as NCT06615206 is an interventional study, until now there is not results resisted, utilized HG204 is a CRISPR RNA-editing to treat MECP2 duplication syndrome. Not completed NCT06025032 early phase I the objective of the trial is to ascertain the safety and efficacy of HG205 as a CRISPR–Cas13 RNA base-editing therapy for treating hearing loss attributed to the p.Q829X mutation in the *otoferlin (OTOF)* gene, NCT05740761 this trial is observational type utilizing CRISPR–Cas9-based gene editing combined with AAV-based delivery for correcting of the *common methyl-CpG-binding protein 2 (MeCP2)* mutations both *in vitro* and *in vivo*. Other trails had conducted on neurological diseases NCT05683860 this is an open-label extension study designed to assess the safety, tolerability, pharmacokinetics, pharmacodynamics, and clinical effects of WVE-004 in adult patients diagnosed with ALS, frontotemporal dementia (FTD), or a mixed ALS/FTD phenotype possessing a confirmed mutation in the *C9orf72* gene. Patients must have successfully completed the Phase 1b/2a WVE-004-001 study to participate in the research. NCT05032196 this is a Phase 1b/2a multicenter, randomized, double-blind, placebo-controlled trial designed to assess the safety, tolerability, pharmacokinetics, and pharmacodynamics of WVE-003 in adult patients with early-manifest HD who possess the specific SNP. NCT04931862 is a Phase 1b/2a multicenter, randomized, double-blind, placebo-controlled trial designed to assess the safety, tolerability, pharmacokinetics, and pharmacodynamics of intrathecal WVE-004 in adult patients with C9orf72-associated ALS or FTD. Patients must possess a documented mutation (GGGGCC [G4C2] repeat expansion) in the initial intronic region of the C9orf72 gene and be diagnosed with ALS or FTD to participate in the study.

## 6 Future directions and emerging trends

CRISPR–Cas9 exhibits considerable potential; yet, its recent discovery and implementation in humans limit its utility in clinical investigations. The utilization of CRISPR–Cas in neurodegenerative diseases presents numerous significant hurdles, each necessitating inventive solutions. Comprehending CRISPR–Cas9 delivery systems. CRISPR–Cas has developed significantly within a relatively short period. The technology has facilitated numerous achievements, and there are elevated prospects for additional advancements in the forthcoming years (247). Current advancements in neurodegenerative disease therapies utilizing CRISPR–Cas demonstrate encouraging developments, particularly in the pursuit of safer and more efficacious molecular interventions aimed at mutant genes associated with these disorders. These advancements include enhanced editing tools such as Cas9 and CRISPR diagnostics variations, as well as novel techniques, including base and prime editing for comparatively accurate gene alterations. These precision procedures are crucial for neurodegenerative disorders, as even minor inaccuracies can have significant consequences due to the brain's functioning (358).

Several intriguing avenues may expedite translation in the future. CRISPR-based diagnostics, such as specific high-sensitivity enzymatic reporter locking (SHERLOCK) and DETECTR, provide rapid and sensitive identification of disease biomarkers and may enhance therapeutic platforms for patient stratification and therapy response monitoring (359, 360). AI-assisted sgRNA design and machine learning models for forecasting off-target effects, structural variant risk, and cell type specificity can enhance the safety and efficacy of next-generation editing (361). Progress in patient-derived organoids of the brain and assembloids facilitates mechanistic investigations of intricate neurodegenerative processes, encompassing neuronal–glial interactions and circuit-level dysfunction, while offering scalable platforms for the validation of CRISPR therapeutics prior to animal and human trials (362, 363).

### 6.1 Treatment of genetic disorders

Although CRISPR–Cas possesses considerable promise for the treatment of various genetic disorders, issues concerning off-target effects and the efficient delivery of the CRISPR–Cas genome editing apparatus must be resolved to facilitate its implementation in clinical settings, particularly for neurodegenerative diseases. The primary problem in creating theragnostic for brain injury is site-specific delivery. Instances have arisen throughout the years in which the transport of nanoparticles (CRISPR–Gold, magneto-electric nanoparticles) has been effectively utilized (303, 364). This technique facilitates genomic modifications, encompassing the deletion of extensive nucleotide sequences, homologous recombination, insertion/deletion point mutations, and transcriptional regulation of certain genetic regions. Owing to its functional characteristics and resilience to epigenetic alterations, CRISPR–Cas9 has emerged as the most advantageous method for genome editing in human disease models, both *in vivo* and *in vitro* (365). Nonetheless, as previously discussion, the primary barrier of effective gene therapy for neurodegenerative diseases is the inadequate comprehension of its pathogenic mechanisms. Nonetheless, with a growing quantity of research aimed at elucidating the molecular causes and formulating gene therapies for this disease indicates that CRISPR–Cas9 technology has considerable promise for enhancing our comprehension of NDs and for effective future interventions targeting specific genes.

### 6.2 Future applications

Future applications of CRISPR–Cas focus on precise, allele-specific editing to selectively target mutant alleles responsible for autosomal dominant NDs such as HD and fAD. Advancements in base editors and prime editing facilitate single-nucleotide modifications without inducing double-stranded breaks, hence reducing off-target effects and enhancing safety profiles (54). In preclinical models of HD, the disruption of the *mHTT* gene with CRISPR–Cas9 has shown promising results, including improved motor performance and reduced neurotoxicity (186, 366).

### 6.3 Current findings to potential clinical translation

Recent preclinical studies indicate that genome- and transcriptome-targeted CRISPR approaches can significantly alter ALS biology *in vivo* and in human cells. Specifically, *in vivo* base-editing or nuclease techniques that reduce mutant *SOD1* expression have demonstrated functional advantages and prolonged survival in murine models, thereby providing robust therapeutic proof-of-concept for gene-specific familial ALS (233). Similarly, the excision of the C9ORF72 GGGGCC repeat expansion via Cas9 diminishes RNA foci and DPRs in cellular and murine models, whereas RNA-targeting CRISPR (Cas13/CasRx) can reduce toxic repeat RNAs/DPRs without modifying DNA providing a complementary, less-permanent approach for patients (228).

Implementing these advancements in clinical settings will necessitate concurrent advancements in four domains. The primary obstacle is the secure and effective delivery to motor neurons (spinal cord and pertinent brain regions) at a therapeutic scale: innovative AAV capsids and non-viral methods (LNPs, nanoparticles, intrathecal or intraparenchymal regional techniques) must be refined for biodistribution, dosage, and immunogenicity in large animals prior to human trials (367). Secondly, it is imperative to minimize genotoxic risk by incorporating base and prime editors that circumvent double-strand breaks, utilizing transient delivery formats (such as RNPs, self-inactivating vectors, and split-intein systems), and conducting thorough, unbiased genome- and transcriptome-wide off-target profiling within preclinical packages (233).

Third, the clinical translation is enhanced by established regulatory and biomarker precedents: the approval and regulatory experience with antisense oligonucleotide therapy for SOD1-ALS (tofersen) illustrates that reducing a disease protein and exhibiting biomarker effects (e.g., neurofilament light) can facilitate accelerated pathways while long-term clinical benefits are validated in confirmatory studies. This presents three practical implications for CRISPR strategies: (a) initial trials should incorporate robust molecular biomarkers (Cerebrospinal fluid (CSF)/plasma neurofilament and target RNA/protein measurements), (b) patient selection must closely correspond with the genomic target (e.g., *SOD1* or *chromosome 9 open reading frame 72 (C9ORF72)* carriers), and (c) early regulatory engagement in development is essential (368).

### 6.4 Challenges

One of the biggest challenges still is effective and focused *in vivo* distribution of CRISPR components. Developed to improve transport over the BBB to reach particular neural populations are innovations include AAV vectors, LNPs, and modified exosomes (224, 369–371). To fix mutations in *SOD1* in ALS mice, for instance, a dual AAV system has been employed to deliver CRISPR–Cas9, hence displaying longer longevity (232). Furthermore, in development are ligand-conjugated nanoparticles to increase CNS cell-type selectivity (372).

CRISPR/dCas9, in conjunction with transcriptional repressors or activators, facilitates epigenetic modulation of gene expression without cleaving DNA. This approach offers a reversible and perhaps safer alternative, especially for multifactorial conditions like PD or sporadic AD. Research has demonstrated that in PD models, dCas9-mediated activation of neuroprotective genes (e.g., *BDNF*) can preserve dopaminergic neurons (373).

The advancement of human-relevant disease models is being revolutionized by CRISPR–Cas technology. Modifying disease-related mutations in iPSCs enables them to emulate the pathogenic characteristics of neurodegeneration. Novel therapeutic targets and disease development regulators are being identified using genome-wide CRISPR screening (374). These tools facilitate the identification of significant genetic associations and cellular pathways involved in disease mechanisms.

The integration of CRISPR-based methodologies with other treatment strategies such as RNA interference (RNAi), antisense oligonucleotides (ASOs), and small molecule pharmaceuticals is a significant trend. This combination may mitigate CRISPR-induced risks while simultaneously enhancing therapeutic efficacy. In AD models, targeting both *APP* and *BACE1* genes has demonstrated a synergistic reduction in β-amyloid levels (375, 376).

## 7 Conclusion

CRISPR–Cas technology possesses the capacity to transform the treatment of neurodegenerative illnesses through precise, targeted, and adaptable modifications to the genome. Preclinical research indicates that it may rectify deleterious mutations, attenuate detrimental gene expressions, and restore cellular function in conditions such as AD, PD, HD, and ALS. Furthermore, CRISPR-based diagnostic instruments and iPSC-derived models have significantly enhanced our understanding of disease mechanisms. Nonetheless, transforming these advancements into practical therapies is exceptionally challenging, particularly regarding their passage through the BBB, minimizing off-target genetic consequences, and stimulating the immune system. Ethical considerations and long-term safety concerns must be addressed for germline editing. Addressing these issues necessitates a multidisciplinary strategy encompassing biomedical innovation, novel drug delivery methods, and governmental oversight. As the science advances, it will be crucial to develop next-generation CRISPR tools, including base and prime editors, high-fidelity Cas enzymes, and RNA-targeting systems, to progress toward safe and effective therapeutics. Ultimately, CRISPR–Cas represents a significant scientific breakthrough, offering potential for the development of novel therapies for now incurable neurodegenerative diseases.

CRISPR-based genome editing is transforming approaches to comprehend and address NDs by the precise alteration of genes and pathways linked to these conditions. Recent advancements underscore the potential of integrating high-fidelity CRISPR systems with AI-optimized gRNA to improve specificity and therapeutic safety. Concurrent advancements in patient-derived organoid models offer more physiologically pertinent platforms for evaluating gene-editing results and forecasting clinical behavior. Advancing the area necessitates the incorporation of CRISPR diagnostics, scalable delivery methods, and comprehensive ethical frameworks to convert these technologies into safe and effective medicines. Collectively, these advancements highlight a dramatic path toward individualized, mechanism-driven therapies for intricate NDs.
